# Don’t Be Surprised When These Surprise You: Some Infrequently Studied Sphingoid Bases, Metabolites, and Factors That Should Be Kept in Mind During Sphingolipidomic Studies

**DOI:** 10.3390/ijms26020650

**Published:** 2025-01-14

**Authors:** Alfred H. Merrill

**Affiliations:** School of Biological Sciences and The Petit Institute for Bioengineering and Biosciences, Georgia Institute of Technology, Atlanta, GA 30332, USA; al.merrill@biosci.gatech.edu

**Keywords:** sphingolipidomics, lipidomics, sphingoid bases, sphingosine 1-phosphate, ceramides, phosphosphingolipids, glycosphingolipids, glucosylceramides, galactosylceramides, LIPID MAPS

## Abstract

Sphingolipidomic mass spectrometry has provided valuable information—and surprises—about sphingolipid structures, metabolism, and functions in normal biological processes and disease. Nonetheless, many noteworthy compounds are not routinely determined, such as the following: most of the sphingoid bases that mammals biosynthesize de novo other than sphingosine (and sometimes sphinganine) or acquire from exogenous sources; infrequently considered metabolites of sphingoid bases, such as N-(methyl)_n_-derivatives; “ceramides” other than the most common N-acylsphingosines; and complex sphingolipids other than sphingomyelins and simple glycosphingolipids, including glucosyl- and galactosylceramides, which are usually reported as “monohexosylceramides”. These and other subspecies are discussed, as well as some of the circumstances when they are likely to be seen (or present and missed) due to experimental conditions that can influence sphingolipid metabolism, uptake from the diet or from the microbiome, or as artifacts produced during extraction and analysis. If these compounds and factors are kept in mind during the design and interpretation of lipidomic studies, investigators are likely to be surprised by how often they appear and thereby advance knowledge about them.

## 1. Introduction

Mass spectrometry (MS) has been an important contributor to the rapid pace of discoveries about sphingolipids over the past few decades. Sphingolipidomic MS analyzes large numbers of compounds using methodologies that are either (and sometimes both) targeted—i.e., looking for features such as precursor–product pairs that are characteristic for particular compounds (for example, see ref. [1,2,3])—or untargeted, wherein all of the detected ions are matched to known or theoretical compounds in lipidomics databases [4,5,6,7]. To improve the accuracy of the identifications, both approaches are often combined with separation methods that utilize other properties of the compounds, such as liquid chromatography [8,9] or ion mobility MS [10], which has been especially helpful for glycosphingolipids [11] since they present so many structural isomers [12].

**Figure 1:**
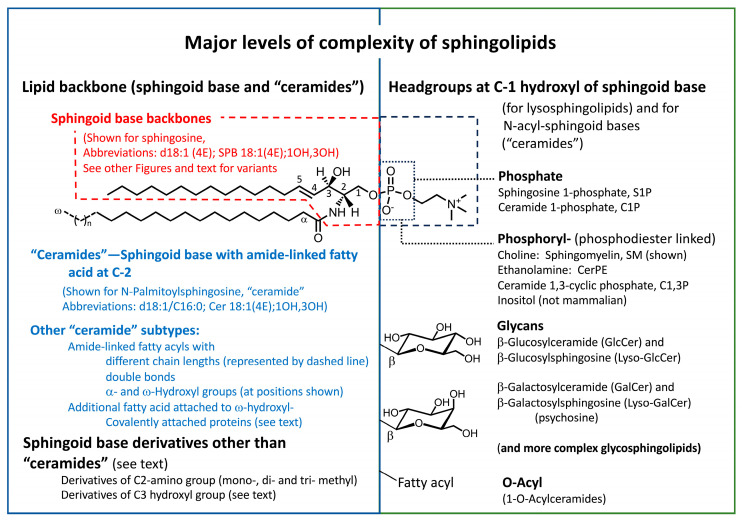
Major levels of complexity of sphingolipids. Sphingolipids are defined as having a sphingoid base backbone, shown here for sphingosine within the red dashed box, which is often given the listed shorthand abbreviations. The first level of complexity for sphingolipids is variation in the structures of the sphingoid base. Derivatives of single functional groups of the sphingoid base represent a second level, namely, (a) addition of fatty acyls to the C-2 amino group for “ceramides” (used as a generic term here for any N-acyl-sphingoid base) with the types of variations listed in blue); (b) addition of a headgroup at the hydroxyl on carbon 1 of the sphingoid base (for sphingosine 1-phosphate and other headgroups, which are often referred to as “lyso-” sphingolipids); and (c) addition of other moieties to the C-2 amino group or C3-hydroxyl as indicated. Complex sphingolipids have a “ceramide” backbone and the headgroup subcategories listed in the right half of the figure.

Nonetheless, the sphingolipidome is comprised of amazingly diverse and complex structural elements, as displayed in the subcategories in Figure 1 and other figures in this review.

Therefore, despite the sophistication of the methods currently available for sphingolipid analysis, many subspecies are not routinely analyzed in sphingolipidomic studies—much less in broader “lipidomic” studies. Omissions will be eventually sorted out as the methodologies improve, but in the meantime, it is worthwhile to keep the infrequently analyzed compounds in mind with respect to where they fit in the “big picture” of sphingolipid metabolism so they can be intentionally looked for and recognized when they might unexpectedly appear.

This review presents an overview of structural variation in (mainly) the lipid backbones of sphingolipids with a few comments on more complex metabolites. When known, the biosynthetic origins and relationships to key branch points will be noted since they will be useful in interpreting lipidomic data. The review also discusses some surprising circumstances where experimental conditions, such as cell culture components and time in culture, as well as some physiological variables, have been noted to cause (originally surprising) shifts in sphingolipid composition. It is hoped that by looking at the sphingolipidome from this perspective, investigators will find it easier to make new discoveries from otherwise puzzling sphingolipidomic data.

## 2. A Brief Overview of Sphingolipid Metabolism

**Figure 2:**
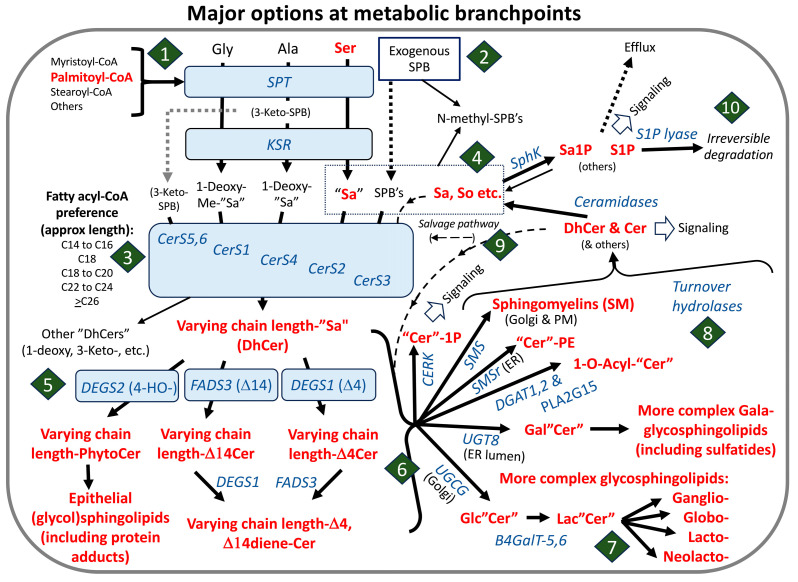
Major options at metabolic branch points. In this depiction, the traditional pathway for sphingolipid biosynthesis de novo begins in the upper left corner (diamond #1 in Figure 2), where serine palmitoyltransferase (SPT) utilizes serine and palmitoyl-CoA to make 3-ketosphinganine (a 3-keto-sphingoid base, 3-Keto-SPB), which is reduced to sphinganine (Sa). Note that these substrates and intermediates for the production of “typical” sphingolipids are indicated with red font throughout this figure. “Atypical” SPB are produced when SPT uses glycine (Gly) as an alternative substrate to make 1-deoxymethyl-Sa or alanine (Ala) to make 1-deoxy-Sa. Sphingoid bases also enter the pathway from exogenous sources (labeled “Exogenous SPB”) (diamond #2) or recycling of SPB from sphingolipid turnover (diamonds #4, 8, and 9). SPB from all of these origins can undergo N-acylation (at the C-2 amino group) by ceramide synthases (CerS) (diamond #3) that have different fatty acyl-CoA chain-length preferences (as listed) and control the type of N-acyl chain in the product “dihydroceramides” (DhCer). Or, SPB can be phosphorylated by sphingosine kinases (SphK) (diamond #4) for cell signaling of irreversible degradation (diamond #10). In some cases, SPB have been found to be N-methylated. Diamond #5 displays the reactions that can convert DhCer) into ceramides (N-acyl-(4E)-sphingosines, Cer) by DhCer desaturase 1 (DEGS1), phytoceramides by DEGS2, or N-acyl-(14Z)-”sphingosines” via FADS3. The action of DEGS1 then FADS3 (or FADS3 then DEGS1) produces the sphingadienine-containing “ceramides”. For more complex sphingolipid biosynthesis, these enter another important branch point (diamond #6), where specific headgroups are added to make sphingomyelins (SM), ceramide phosphoethanolamines (CerPE), 1-O-acylceramides (1-O-Acyl-“Cer”), galactosylceramides (GalCer), glucosylceramides (GlcCer), and ceramide 1-phosphates (Cer1P) (for more specifics, see [13]). GlcCer and GalCer can be metabolized to more elaborate glycosphingolipids with additional carbohydrates, which are divided into root structures based on the next added sugars (diamond #7) and other functional groups, such as sulfate (for sulfatides). Sphingolipid turnover is also a complex process (diamond #8) with multiple hydrolases and subcellular locations. The resulting DhCer and Cer can participate in cell signaling (as precursors for “Cer” 1-phosphates, “Cer”-1P), recycled into complex sphingolipids (diamond #9), or hydrolyzed to sphingoid bases, which can be recycled or phosphorylated, as shown in diamond #4. Besides participating in cell signaling, sphingoid base 1-phosphates can be irreversibly degraded to a fatty aldehyde and ethanolamine phosphate (diamond #10).

The scheme in Figure 2 summarizes the major steps in de novo sphingolipid biosynthesis and turnover, plus the utilization of exogenously provided sphingoid bases and recycling (versus degradation) of some of the intermediates.

The presentation of sphingolipid biosynthesis in this manner (Figure 2) accounts for the production of not only “traditional” sphingolipids (Figure 1), but also the so-called “atypical” sphingolipids (e.g., 1-deoxysphingolipids) and the utilization of sphingoid bases from recycling or exogenous sources, which will be useful to know in the discussions of such compounds in the rest of this review.

Some important points that are worth bearing in mind are as follows: (1) sphingoid bases can arise from multiple routes—de novo biosynthesis from serine (and additional amino acids) (diamond #1 of Figure 2), recycling of sphingoid bases from cellular turnover (diamond #9 of Figure 2), and uptake from exogemouis sources (“Exogenous SPB” in Figure 2); (2) sphinganine and other SPB are mainly N-acylated by a family of ceramide synthases (CerS) that control the type of N-acyl chain by their fatty acyl-CoA preference (diamond #3), but they can also be phosphorylated by sphingosine kinases (SphK) (diamond #4); (3) DhCer can undergo desaturation or hydroxylation of the sphingoid base backbone (diamond #5) by the enzymes shown (DEGS1, DEGS2, and FADS3) *or* be incorporated into more complex dihydrosphingolipids (shown by the bracket at diamond #6), which is also the fate of most unsaturated or hydroxylated “ceramides”; (4) headgroup addition (diamond #6) is a key branch point of complex sphingolipid biosynthesis that is controlled not just by the enzymes that catalyze addition of the specific headgroup (for example, phosphosphocholine for sphingomyelin via SM synthases, SMS) but also by their transport from the endoplasmic reticulum (ER) (where “Cer” is made) to the subcellular localizations of the enzymes by both vesicular trafficking and Cer transport proteins (CERT). These locations are primarily the *trans*-Golgi and plasma membranes for SMS, the *cis*-Golgi for GlcCer synthase (UGCG), the lumen of the ER for GalCer synthase (referred to as UGT8 or CGT) and possibly SMSr, which makes CerPE. A more in-depth discussion of these processes (and of more complex glycosphingolipid biosynthesis) can be found in other reviews of the metabolic pathway [13,14,15,16].

Sphingolipid turnover is also a complex process (diamond #8 of Figure 2) with multiple hydrolases in more than one location (e.g., lysosomes for housekeeping turnover and the plasma membrane and other organelles for signaling). The resulting DhCer and Cer can participate in cell signaling directly and by serving as precursors for “Cer” 1-phosphates, “Cer”-1P) and sphingoid bases that can be phosphorylated, as shown in diamond #4, for signaling or as intermediates for irreversible degradation (to fatty aldehydes and ethanolamine phosphate by S1P lyase (diamond #10). “Cers” and SPB can also be “salvaged” for reincorporation into complex sphingolipids (diamonds #4 and #9).

Sphingolipids are found in all mammalian tissues and display interesting differences among plasma, skin, organs, and even between cell types within an organ (for the most comprehensive sphingolipid analysis to date, for murine tissues, see [17]). In general—but not always—compositional differences are reasonably associated with the related branch point enzymatic activities (for example, the N-acyl-chains of the Cer backbones are related to the CerS subtypes that are present); however, there are enough differences for lipidomic analyses to be essential in identifying what sphingolipids are present. In addition, many factors have been found to perturb sphingolipid metabolism [18], and some of these will be covered in this review from the perspective of how an investigator might stumble on variants of the major sphingolipid subspecies among the ions identified from a sphingolipidomic analysis.

## 3. Well-Known and Less or Infrequently Analyzed Sphingoid Bases

Structural diversity begins with the sphingoid base backbone, which is intrinsic to the biochemical properties of serine palmitoyltransferase (Figure 2, diamond #1), which has been called a “promiscuous enzyme” due to the range of substrates that it can utilize [19]. Sphingolipidomic analyses of samples from mammals usually give emphasis to sphingosine and its many metabolites (Figure 1) since they are the predominant subspecies in most samples. They also have the longest history, being initially described (and named “sphingosin”) by Thudichum in 1881 [20], assigned the 18-carbon-chain length by Klenk in 1929 [21] and the full structure by Carter and co-workers in 1947 [22], who proposed that compounds in this family should be termed sphingolipids [23]. Hundreds of different sphingoid base variants are now known for eukaryotes and bacteria [24,25,26], and while much has been learned about some of these compounds, it is still reasonable to state, as Karl-Anders Karlsson did in 1970 [27], that “There may thus be a relation between complexity of tissue functions and base composition of a sphingolipid. However, before a functional characterization of long-chain base derivatives may be done, there remains a considerable amount of work on the chemistry and occurrence of the long-chain bases”.

### 3.1. Sphingoid Bases Made De Novo by Mammals

**Figure 3:**
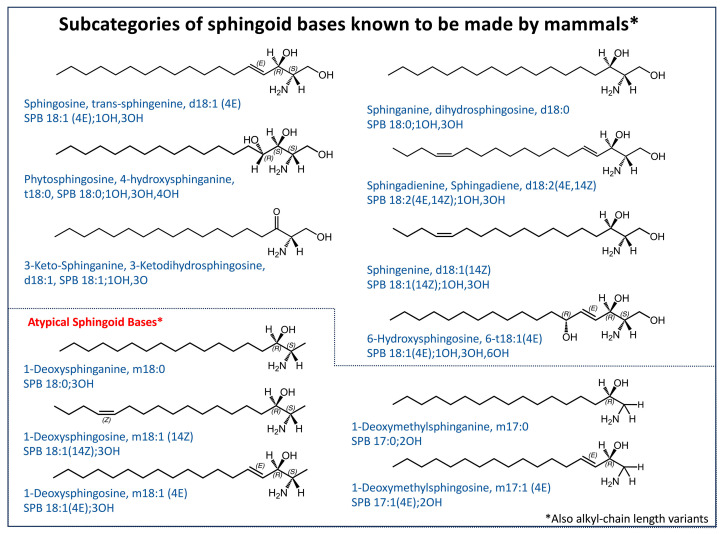
Subcategories of sphingoid bases known to be made by mammals. Each of these sphingoid bases has been found to be made de novo by mammals. All in the upper subsection of the figure are made from serine; those in the lower subsection are made from alanine (left column) and glycine (right column). Only the chain length from palmitoyl-CoA (e.g., a total of 18 carbons for typical sphingoid bases and 1-deoxysphingoid bases, and 17 for 1-deoxymethylsphingoid bases) are shown, but other chain lengths are also produced by SPT under special circumstances discussed in Section 3.1.1.

Sphingoid bases vary in chain length, number of double bonds, number of hydroxyls, and other features. Before discussing them, it is worthwhile to comment on nomenclature and abbreviations. Sphingosine is the generally accepted name for the specific compound discussed above, but it is also referred to as (4E)-sphingenine or by an abbreviation such as d18:1(4E). This nomenclature uses “d” to represent the two hydroxyls (if there is only one, it is labeled “m” for mono-, and three are designated “t” for tri-), “18:1” reflects the 18-carbon chain and one double bond, and “4E” describes the *trans* double bond between carbons 4 and 5. A modification of these abbreviations has been suggested by LIPID MAPS [28], wherein the subtype is given (SPB for sphingoid base), followed by the carbon number and double bonds (with stereochemistry) and location of other functional groups (i.e., SPB 18:1(4E);1OH,3OH for sphingosine). By these schemes, if the alkyl chain is fully saturated, the compound can be named dihydrosphingosine or sphinganine (the IUPAC recommendation) and abbreviated d18:0 or SPB 18:0;1OH,3OH (Figure 3). When there are two double bonds, the usual name is sphingadienine (sphingadiene is also sometimes used) with the abbreviation designating the extra double bond and type (for mammals, the additional double bond is 14Z (*cis*), and the two most frequently used abbreviations are d18:2(4E,14Z) and SPB 18:2(4E,14Z);1O,3OH), as shown in Figure 3). If the sphingoid base has a third hydroxyl at position 4 (and no double bonds), the simplest abbreviation is t18:0 (for mammals, the major compound in this category is called 4-hydroxysphinganine, and often colloquially “phytosphingosine” even though the backbone does not contain the 4E-double bond of sphingosine). These nomenclatures can be a source of confusion because the skin contains substantial amounts of a trihydroxy-containing sphingoid base with a 4E-double bond (t18:1), however, the hydroxyl is at the sixth carbon (6-hydroxysphingosine, 6-hydroxy-t18:1), as shown in Figure 3. This type of ambiguity is resolved by using the LIPID MAPS abbreviation (SPB 18:1(4E)1OH,3OH,6OH) [28].

**Figure 4:**
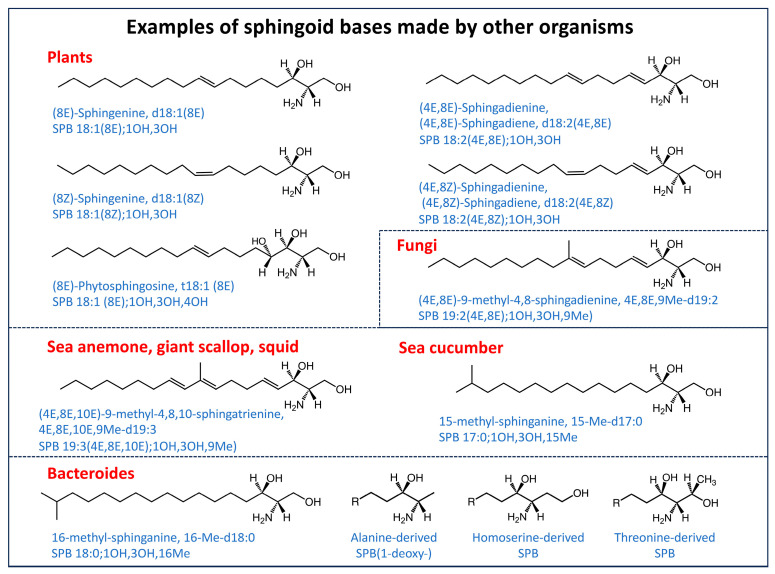
Examples of sphingoid bases made by other organisms. These sphingoid bases have been found to be produced by the organisms named in red font as well as many additional organisms (but not humans). See the text for references to these compounds.

Complications can arise whenever samples contain isomers with the same molecular composition—for example, mammalian sphingoid base d18:1(4E) versus d18:1(8E and 8Z), which are found in plants [29,30] (cf., Figure 4 that is in Section 3.2)—but the exact structure has not been definitively established by the method that was used for analysis. For clarity as well as to allow data to be more accurately deposited into databases, LIPID MAPS has recommended that lipidomics data use an abbreviation system that includes the level of certainty of the assigned structure [28]. This system modifies the older abbreviation scheme for sphingoid bases (SPB) (as well as other lipids, see [28]) by specifying the positions of the hydroxyls and other features when they are fully characterized (e.g., sphingosine is SPB 18:1(4E);1OH,3OH if definitively identified), but would be given the less specific designation “SPB 18:1;O2” if only known with certainty at the level of its molecular composition. This nomenclature is also applied when the sphingoid base is incorporated into more complex structures [28]. The rest of this review will describe findings from the literature that have not always fulfilled these rigorous criteria, but sphingolipid researchers should be mindful of this issue as they read lipidomics reports.

#### 3.1.1. Chain Length Variants

Although the chain length of the sphingoid bases of most mammalian sphingolipids is 18 carbons due to the preferential utilization of palmitoyl-CoA by the major isoenzymes of serine palmitoyltransferase (SPTLC1 and SPTLC2) (reviewed in [15]), chain lengths from 12 to 32 carbons have also been found, with some of the greatest variation in skin [24,31]. Sphingoid bases with these other chain lengths are infrequently analyzed even though they have been known to exist for over a half-century. This is probably due to their usually low abundance and, until recently, insufficient knowledge about their origin and impact on biological function. The 16- and 20-carbon chain length sphingoid bases are made de novo by different combinations of the subunits of serine palmitoyltransferase (SPT), which is a trimeric complex of two large subunits (SPTLC1 and SPTLC2 or SPTLC3) and a small subunit (SPTssa or SPTssb). Expression of SPTLC3 results in the favored use of myristoyl-CoA and production of 16-carbon-sphingoid bases [32,33], and expression of SPTssb promotes the use of stearoyl-CoA and production of 20-carbon sphingoid bases [32,34]. The d16:1(4E)-containing sphingolipids have been found in tissues expressing SPTLC3, such as the heart [35], and have been implicated in ischemic cardiomyopathy [35,36], renal cell cancer [37], and gastric cancer [38], and are found in human plasma [39]. Sphingolipids with d20:1(4E) are also found in human plasma [39], skin [40], the eye [41], various cancers [42], and especially in the brain [43], where the longer backbone influences the membrane properties [44] and differential localization of gangliosides [45,46], and increases with aging [47] but the ratio of d20:1 to d18:1 in GM1 decreases with Alzheimer’s disease [48]. Elevation of 20-carbon long-chain bases due to a mutation in SPTssb results in neurodegeneration [34].

Many tissues [24,25,26] (especially skin [31]) have been noted to contain small but detectable amounts of other sphingoid bases, including odd-carbon number sphingoid bases that are linear or branched (branched sphingoid bases that are comprised of an ω-2 methyl-group are termed “iso”, and those with an ω-3 methyl group are called “anteiso”). These might arise from the accommodation of a broader spectrum of fatty acyl-CoAs by some SPT isoenzymes [49] and/or reflect sphingoid bases that have been taken up from exogenous sources since branched sphingoid bases are more common in the sphingolipid metabolic pathways of other organisms [15,50,51].

#### 3.1.2. Double Bond Variants

Two double bond variants are well known in mammals but somewhat less frequently analyzed than sphingosine: sphinganine, which lacks any double bonds and is distributed as widely as sphingosine because it is a biosynthetic precursor to sphingosine via dihydroceramides (which are distinctly different from ceramides in biophysical properties and functions) [52,53,54,55], and (4E,14Z)-sphingadienine (sphingadiene), which is produced by the addition of a *cis*-(Z) double bond to the sphingosine of ceramides by FADS3 [56] (Figure 2). (4E,14Z)-Sphingadienine has been known for a long time to be a major component of plasma sphingolipids [57,58] (estimated to be as high as 18% of the total) [39]. It is present in other tissues such as the brain [59,60] and is elevated in Fabry disease [61]. The 14-*cis* double bond greatly alters its membrane properties and functions [24,25,26].

FADS3 is able to introduce a 14Z-double bond directly into DhCer [56,62], thereby forming isomers of traditional sphingosine and ceramides, but appears to be mainly involved in the biosynthesis of (4E,14Z)d18:2 unless DEGS1 is defective [63]. Its other substrates include iso-branched-chain Cer [56], 1-deoxyDhCer (but apparently not 1-deoxymethylDhCer) [63,64], and possibly free sphingosine [62], although that is uncertain [56]. Not much has been done to explore if mammals have diene sphingoid bases in other positions, but new MS methods should facilitate those types of analyses [65].

The double bonds of mammalian sphingoid bases have long been thought not to arise from the utilization of unsaturated fatty acyl-precursors by serine palmitoyltransferase since its activity in vitro is essentially undetectable with *cis*-unsaturated fatty acyl-CoAs (the predominant types in mammals), although substantial activity was found using *trans*-9-hexadecenoyl-CoA, presumably due to its steric similarity to the optimal substrate with a saturated alkyl chain, palmitoyl-CoA [66]. This is still thought to be the case under normal circumstances, but interest in *trans*-fat-induced atherogenesis has prompted a recent assessment of whether *trans*-fatty acids can be utilized for sphingoid base biosynthesis in vivo [67], and both mammalian cells in culture (Huh7 hepatocarcinoma cells) and mice were found to incorporate *trans*-unsaturated fatty acids into the respective unsaturated sphingoid bases. For example, elaidate, C18:1(9E)—a *trans*-fatty acid common in vegetable oils hydrogenated by some procedures—was incorporated into (11E)d20:1 and (4E,11E)d20:2. Furthermore, the use of an SPT inhibitor (myriocin) was shown to link sphingolipid biosynthesis and the effects of feeding high-fat diets enriched in *trans*-fatty acids to low-density lipoprotein (LDL)-receptor-deficient mice (increased very-low-density lipoprotein and sphingolipid secretion into circulation and atherogenesis compared with high-fat diets with *cis*-unsaturated fatty acids). The findings of this study provide another justification for verifying the location and stereochemistry of double bonds in sphingoid bases and downstream metabolites when organisms have been exposed to *trans*-unsaturated fatty acids in a laboratory setting or diet [68].

#### 3.1.3. Hydroxyl-Variants

The two major sphingoid bases of mammals with an additional hydroxyl (Figure 3) are phytosphingosine (4-hydroxy-sphinganine) (made from DhCer by DEGS2) [69,70], which is found in skin [71], urinary bladder [72], and many other tissues (including brain) [73,74], and 6-hydroxy-sphingosine [71,75], which is rarely analyzed except in studies of skin. The extra hydroxyl strongly affects membrane order and permeability [76,77], and 6-hydroxy-sphingosine is thought to influence the barrier against water evaporation and lipophilic permeant(s) [78].

#### 3.1.4. 1-Deoxy-Sphingoid Bases (Often Referred to as “Atypical” Sphingoid Bases)

Another subcategory of hydroxyl-variants lacks the hydroxyl at carbon 1 (1-deoxysphingoid bases) or the entire hydroxylmethyl moiety at that position (1-deoxymethylsphingoid bases) (Figure 3). They were discovered during studies of mutant SPTs that cause peripheral neuropathy [79,80] and independently by studies of the consequences of inhibition of ceramide synthase(s) by fumonisins [81], which also revealed that they are present in cells with native SPT but usually not noticed because they are mainly present as N-acylated metabolites rather than the free sphingoid bases. 1-Deoxy- and 1-deoxymethyl-sphinganines are produced by SPT because the native enzyme prefers serine (Ser) but also accommodates alanine (Ala) and glycine (Gly), respectively, although less well [82,83]; and the mutations that cause inherited neuropathies have less of a preference for Ser. Since their production is influenced by any state that affects the availability of these three amino acids, they are presumably always made in some amount but have been overlooked in many sphingolipidomic studies.

Both 1-deoxy- and 1-deoxymethyl-sphingoid bases are initially metabolized in an analogous manner to sphinganine with reduction of the 3-keto-products of SPT, followed by N-acylation by CerS (although there might be differences in the N-acyl-distributions, [84]); however, a distinct difference occurs when the N-acyl-derivatives are desaturated—the major product for 1-deoxyceramides has a 14Z-double bond rather than the 4E-double bond of ceramides and N-acyl-1-deoxymethylceramides [64]. This was determined by a useful gas phase reaction of lipid double bonds with ozone [64,85], although this was later reported to produce lower abundance ions from 4E double bonds due to a more sluggish rate of reaction [85]. Nonetheless, the 14Z-location was confirmed by the formation of the dimethyl disulfide derivatives [64] and by subsequent investigations. Another method that has been reported to distinguish the 4E- and 14Z- isomers of 1-deoxysphingosine is cryogenic infrared spectroscopy [86]. The enzyme that is responsible for the introduction of the 14Z-double bond is FADS3, as occurs in the biosynthesis of the second double bond of sphingadienines [62]. The 4E- and 14Z-isomers are separable by reverse-phase chromatography (14Z eluting earlier), and we have noted (but not published) in LC-MS/MS analyses that despite the predominance of 14Z-desaturation, (4E)-1-deoxyceramides were clearly made in substantial amounts in many cell lines in culture. For example, in one of these unpublished studies, LYB cells (which lack SPT activity) [87] were incubated with 1-deoxysphinganine and then analyzed by LC-MS/MS using reverse phase chromatography, which detected a series of 1-deoxyDhCer and both (4E)- and (14Z)-deoxyceramides; then, the presence of the double bond at 4E- was confirmed by the Lemieux–von Rudloff reaction [88], which produced the expected fatty acyl-alanines. The results of these preliminary studies verify that under certain conditions, the 1-deoxyDhCer can be desaturated at the 4,5-position, presumably by DEGS1 or 2, and that attention should be given to verification the double bond position(s) when analyzing these compounds.

1-Deoxysphingolipids have been associated with numerous pathologic conditions in addition to the original neuropathies [83], including diabetes [89,90], liver disease [91,92], and docetaxel neurotoxicity [93]. There is a possibility that they might be encountered not only from de novo biosynthesis but also from uptake in the gastrointestinal (GI) tract from intestinal microflora and many foods [82,94]. Historically, they were first noted as components of the Arctic surf clam (and named spisulosine/ES-285) that showed promising biologic effects in screens for naturally occurring anti-cancer agents [95]. Thus, although 1-deoxysphingolipids have been mostly associated with deleterious effects, they might also have beneficial physiologic functions. One indication of the latter is the recent report that 1-deoxysphingosines are modulators of the activity of NR2F1 and 2 (COUP-TFs), which are orphan nuclear hormone receptors that are critical for the development of the nervous system, heart, veins, and lymphatic vessels [96]. Likewise, even though they can cause major perturbations of the biophysical properties of membrane bilayers [97,98,99] and skin [100], it is not known if these are physiologically useful or disruptive (or both, depending on the context). These are sufficiently provocative possibilities to include this subcategory of sphingolipids in more sphingolipidomic investigations.

#### 3.1.5. 3-Keto-Sphingoid Bases

These compounds (Figure 2) have long been known to be produced de novo as the first intermediate of sphingoid base biosynthesis (Figure 2), but are rarely analyzed. This is probably because the amounts were below detection in early studies, and the enzyme that reduces the 3-keto group to produce sphinganine, 3-ketosphinganine reductase (KDSR), was found to have ~10-fold higher activity than SPT [101] and only became rate limiting when assays lacked a reduced pyridine nucleotide [66,102]. Nonetheless, early studies also found that 3-ketosphinganine can be N-acylated [103], which left the possibility that there might be conditions where these compounds accumulate and/or are incorporated into more complex sphingolipids. More studies of these compounds are warranted now that MS methods have become available for the analysis of 3-ketosphinganine and downstream metabolites (e.g., 3-ketoDhCer) [104], and the genes for KDSR have been identified in yeast (TSC10/YBR265w) [105], mammals (initially associated with the follicular variant translocation protein 1, FVT-1, then renamed KDSR) [106,107], and other organisms, including bacteria (which provided a metabolic surprise [108,109] that will be discussed shortly).

Many studies have now reported noticeable amounts of 3-keto-sphingoid bases as well as more complex sphingolipids with these backbones. For examples: an analysis of the free sphingoid bases in yeast (wild-type strain BY4741) found comparable amounts of 3-ketosphinganine and sphinganine [110]; 3-ketosphinganine was readily detected in HeLa cells [104,111], and they contained even higher amounts of 3-ketoDhCer; 3-ketoDhCer was also seen in Hek cells that were stably overexpressing SPT and greatly elevated when the cells were incubated with supplemental Ser and palmitate [112]; rat-liver mitochondria have been reported [113] to contain more 3-ketosphinganine in ceramides than sphingosine or sphinganine, and 3-ketosphinganine was also found in glycosphingolipids; 3-ketosphinganine was elevated above sphinganine in plants with abnormal flux through the de novo biosynthetic pathway [114]; LLC-PK1 cells were found to have elevated 3-ketosphinganine when treated with a sphingosine kinase inhibitor [115]; and 3-ketoceramides have been found in analyses of sphingolipids from *Caulobacter crescentus* [108]. These instances make it clear that these compounds are not as rare as originally thought.

One of the reasons that they have been overlooked is that it is not widely appreciated that 3-ketosphingoid bases are N-acylated under some circumstances (and 3-keto-DhCer can be mistaken for Cer), such as when 3-ketosphinganine is produced too rapidly [112] or in large amounts (e.g., when KDSR activity is low or absent). Another cause—the metabolic surprise referred to above [108]—has been recently found with *Caulobacter crescentus*, a bacterium that thrives in nutrient-poor freshwater lakes and streams [116]. Instead of reduction of 3-ketosphinganine to sphinganine followed by N-acylation to produce DhCer, as occurs in other organisms, *C. crescentus* first acylates 3-ketosphingainne to 3-keto-DhCer, which are subsequently reduced to DhCer by a ceramide reductase (CerR) [108]. The pathway operates differently because only two of the enzymes of the pathway, SPT and CerS, are located in the cytoplasm, whereas 3-keto-reductase (CerR) is in the periplasmic space [109].

Mutations in KDSR also elevate 3-ketosphinganine(s) and have been found to result in multiple dermatologic abnormalities, including varying degrees of progressive symmetric erythrokeratoderma and additional disorders of keratinization plus a reduction in the number of blood platelets (thrombocytopenia) [117,118,119,120,121,122]. Global metabolic profiling and LC-MS analyses found that 3-ketosphinganine was highly elevated in plasma from patients with mutations in KDSR compared to controls [120], but there was no reduction in downstream sphingolipids in the patients, with the authors suggesting this might indicate compensation of defective sphingoid base biosynthesis by salvaging of preexisting sphingolipids [120]. An in-depth MS analysis of the sphingolipids of two patients with compound heterozygous KDSR mutations [117] found that the elevated 3-ketosphinganines had alkyl chain lengths that ranged from 18- to 26-carbons, and substantial portions were N-acylated.

A missense mutation in KDSR (kdsrI105R) found in zebrafish results in loss of function and progression of hepatomegaly to steatosis, then hepatic injury [123]. These investigators also found evidence for increased salvaging of sphingolipids and expected that 3-keto-sphingolipids probably accumulated, although they were not analyzed. The liver cells displayed aberrant mitochondrial morphology, oxidative stress, and ER stress. There was elevated expression of sphingosine kinase 2 (sphk2), and sphk2 depletion suppressed the liver defects, which suggests that they might be mediated by a phosphorylated sphingoid base.

Interesting connections between 3-keto-sphingolipids and cancer have also been found. An investigation of the expression of FVT-1 (KDSR) in B-cell non-Hodgkin lymphomas [124] found that FVT1 expression by follicular lymphoma did not differ significantly when compared with control tonsil and other types of low-grade B-cell lymphoma (despite the original naming of this gene as a “follicular lymphoma variant”); however, cancer subtypes with lower expression of FVT-1 were correlated with longer survival [124]. This is suggestive that accumulation of 3-keto-sphingolipids might be cytotoxic, and exogenous addition of 3-ketosphinganine has been found [125] to be cytotoxic for HGC27 cells (human gastric carcinoma), U87MG cells (malignant glioma), and T98G cells (glioblastoma) and to induce autophagy (although these effects did not seem to be due to 3-keto-sphingolipids per se because they were not elevated in the cells) [125]. In another study, exogenously added 3-ketosphinganine was cytotoxic for MV4-11 (biphenotypic leukemia) and MOLM13 (AML) cells, and these cells were also affected by knockout of KDSR, which elevated intracellular 3-ketosphinganine [126]. Loss of KDSR led to apoptosis, cell cycle arrest, and abnormalities in ER structure that were attributed to defects in the unfolded protein response [126]. Leukemias appear to be particularly sensitive because similar effects were seen for KDSR knockout in two additional blood cancer types, Jurkat (T-ALL) and Hut78 cells (T cell lymphoma), but not for four other cell types, 293T cells (transformed embryonic kidney), U251 cells (glioblastoma), SW620 (colon cancer), and A673 (Ewing’s sarcoma).

An additional factor that influences the toxicity of endogenously made 3-ketosphinganine is the activity of SPT, as it determines the rate of production, thereby influencing the level of accumulation of this intermediate. A study [127] of the effects of CRISPR-Cas9-mediated disruption of SPT and KDSR in 12 cancer cell lines found that disruption of KDSR resulted in a greater than 40% reduction in the viability of DLD1 (colorectal adenocarcinoma), HT1080 (fibrosarcoma), NCIH838 (lung adenocarcinoma), U251MG (astrocytoma), MDA-MB-231 (breast adenocarcinoma), and A549 (lung adenocarcinoma) cells (termed “sensitive”) but not the “insensitive” lines DU145 (human prostate cancer), HUH7 (hepatocellular carcinoma), LN229 (glioblastoma), PA16C (pancreatic ductal adenocarcinoma), MDA-MB-415 (breast adenocarcinoma), or COLO205 (colorectal adenocarcinoma), nor a panel of seven “normal” (non-cancer, primary or immortalized) cell lines of varying tissue origin. In the sensitive cell lines, knockout of KDSR induced the formation of irregular, distended subcellular structures and apoptosis (as well as ER stress, shown in later experiments), but not in the insensitive cancer lines and normal lines. MS analysis of the DLD1 cells found that KDSR knockout elevated 3-ketosphinganine > 200-fold when compared to the control or SPTLC1 knockout lines; furthermore, supplementation of the medium with palmitate (a substrate for SPT) caused a further elevation (and greater loss of viability) in the KDSR knockout cells (it does not appear that 3-ketodihydroceramides or other 3-ketosphingolipids were analyzed). Exogenous 3-ketosphinganine was also toxic. Thus, the rate of 3-keto-sphinganine production (and factors that affect it) versus the rate of 3-ketosphinganine reduction (e.g., KDSR activity) influences whether sufficient 3-ketosphinganine is produced to be toxic, and this might be a productive direction for therapeutic intervention. A comparison of the relative levels of mRNA expression for enzymes of sphingolipid metabolism was published in the supplement of reference [128] and a doctoral thesis [111] for the 59 cancer cell lines in the NCI 60-cell line drug screen [129] (NCI-60, http://discover.nci.nih.gov/cellminer/, accessed on 12 December 2024), and there were about a dozen lines from multiple categories (i.e., breast, colon, glioma, leukemia, ovarian, and renal cancer) that appear to have higher expression of SPTLC 1 or 2 but average or lower expression of KDSR. The lowest level of expression of KDSR was found for HeLa cells, which were examined by MS and had easily detectable 3-ketosphinganine and 3-keto-DhCer [104,111].

It should be evident from these examples that 3-keto-sphingolipids are present in a wide variety of conditions and have probably been missed because they have not been looked for.

### 3.2. Sphingoid Base Types Produced by Other Organisms and Potentially Utilized by Animals

In addition to the sphingoid base subspecies that are made de novo by mammals (Figure 3), many organisms produce sphingoid bases with other chain lengths, locations of double bonds, and other features, as illustrated by the examples in Figure 4 [24,26]. Particularly noteworthy from a lipidomics perspective are those that are isomers of mammalian sphingoid bases—for example, plant “sphingosines”, “sphingadienines”, and “phytosphingosines” that have an 8E or 8Z double bond [29,130] (sometimes in addition to a 4E-double bond) and, thus, will not be distinguishable by most analytical methods currently employed, nor definitively identified and/or quantified due to lack of internal standards.

Another interesting subcategory encompasses branched-chain sphingoid bases made by fungi [131,132], sea cucumbers [133], *Bacteroides* [134], and other organisms (Figure 4). The *Bacteroides* SPB shown in Figure 4 includes new subcategories made from homoserine and threonine that were found by novel means [50] that will be discussed later in this review. All of these structural variants (and more described in the references) should be kept in mind during lipidomic analyses because they might (and in some cases already have been found to be) taken up to some extent from food and intestinal microflora [51,133,135,136,137]. 1-Deoxysphingoid bases (Figure 3 and Figure 4) have also been found in many foods, especially seafood and pork [94,138]. For additional information about structural variations found in other organisms, see [24,25,26,29,51,130,139].

## 4. Sphingoid Base Metabolites (Beginning with Derivatives of One Functional Group)

**Figure 5:**
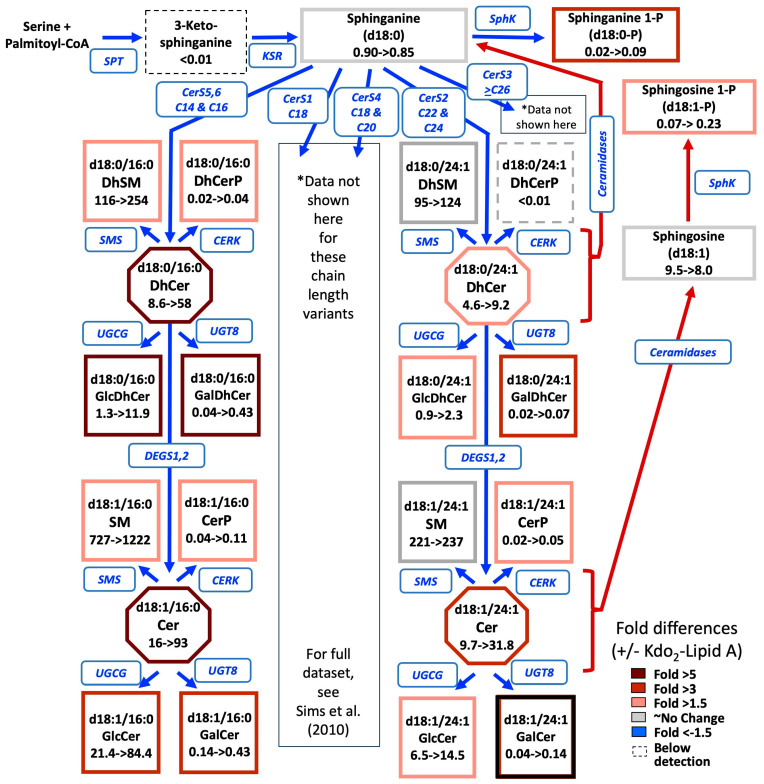
An abbreviated depiction of the production of sphingolipids by RAW264.7 cells after 24 h of incubation with or without Kdo_2_-Lipid A (modified from Figure 1 of reference [140]). As in Figure 2 of this review, de novo biosynthesis begins in the upper left corner to produce sphinganine (d18:0), which is either phosphorylated (to the right) or N-acylated by ceramide synthases (down) that form different chain length dihydroceramides (DhCer), as shown for N-palmitoyl-DhCer (d18:0/C16:0) produced by CerS5 and 6 and for N-nervonoyl-DhCer (d18:0/C24:1), which is produced by CerS2 in most tissues. These may be desaturated by DEGS1 or 2 to make the corresponding ceramides (Cer) (d18:1/C16:0 and d18:1/C24:1, respectively) and/or utilized to make more complex sphingolipids: (Dihydro)SM, (Dihydro)Cer1P, (Dihydro)GlcCer, or (Dihydro)GalCer. Turnover by hydrolases and ceramidases releases the sphingoid bases (sphinganine and sphingosine) that can be recycled or phosphorylated, as shown to the right. The bottom numbers within each enclosure (rectangles for sphingoid bases, octagons for (Dh)Cer, and squares for more complex metabolites) are the millions of molecules per average RAW264.7 cell without (left of the arrow) and with (to the right of the arrows) Kdo_2_-Lipid A. The colors of the outlines reflect the fold differences for +/− Kdo_2_-Lipid A (the color key is shown to the lower right).

As was shown in Figure 2, an important branch point of sphingolipid biosynthesis occurs after the production of sphinganine—its N-acylation by ceramide synthases with different fatty acyl-CoA selectivities versus its phosphorylation. This has also been depicted in the following abbreviated pathway (Figure 5) that not only shows the individual species that are made but also gives the numbers of molecules that have been measured to be present in an average RAW264.7 cell in culture with and without stimulation by the TLR4 receptor agonist Kdo_2_-Lipid A [140].

Sphingoid bases and sphingoid base 1-phosphates (as well as other signaling sphingolipids such as Cer1P) are widely appreciated [141] to be present in much smaller amounts than (Dh)Cer (by several orders of magnitude), which, in turn, are generally less abundant than simple hexosyl(Dh)Cer. The predominant sphingolipids are often the (Dh)SMs. This is also evident in the data in Figure 5, where the cells have on the order of ~0.9 million molecules of sphinganine, ~9 million molecules of sphingosine (and about 10-fold less of the 1-phosphates of these), and considering just these two DhCer and Cer chain lengths (C16:0 and C24:1, which were the most prevalent for these cells), there are at least 10 to 100 million molecules of (Dh)Cer per cell, 1 to 84 million molecules of Glc(Dh)Cer per cell, and ~100 to 1200 million molecules of (Dh)SM per cell. Interestingly, the treatment of the RAW264.7 cells with Kdo_2_-Lipid A increased essentially every subcategory of sphingolipid, which was attributed to the up-regulation of overall sphingolipid biosynthesis, and the total number of sphingolipids increased from 1.5 × 10^9^ to 2.7 × 10^9^ molecules per cell [141].

These sphingolipids that were analyzed in this study that was published 15 years ago [141] are essentially the same ones that are analyzed in most sphingolipidomic studies today (with the exception of GlcCer and GalCer, which are reported as “hexosyl” Cer in most lipidomics studies today). This review will discuss these major subcategories of sphingolipids below but mainly focus on sphingolipids that are infrequently analyzed. The rest of Section 4 will discuss metabolites with a single substituent added to a sphingoid base (at the amine or hydroxyl groups), and the next section (Section 5) will discuss compounds with multiple functional groups (which will be mostly headgroups for “ceramides”). The discussions that follow will not attempt to mention every subspecies that has been seen because the current methodologies rarely look for the more minor or atypical metabolites. Thus, nearly all of the sphingoid bases that were discussed in Section 3 (with the exception of 1-deoxy- which cannot be derivatized at that hydroxyl group) should be kept in mind as potential backbones of more complex sphingolipids.

### 4.1. Sphingoid Bases 1-Phosphates

Sphingoid bases 1-phosphates are widely analyzed despite their frequently low abundance because they are so important in cell signaling, as any reader of this review is undoubtedly already aware. S1P is the most prevalent sphingoid base 1-phosphate, but many tissues also contain sphinganine 1-phosphate and/or phytosphingosine 1-phosphate [74] as well as chain-length variants of these subcategories [142]. Few sphingolipidomic analyses have attempted to analyze a wide spectrum of these compounds, but a recent characterization of the sphingolipids of mouse tissues [17] found multiple sphingoid base 1-phosphates, including d16:1-P, d17:1-P, d18:0-P, d18:1-P, d18:2-P, and d20:1. Some conditions with elevations in some of the less frequently analyzed sphingoid base 1-phosphates are as follows: human cancer patients undergoing treatment with oxaliplatin (d16:1 S1P) [143]; animals consuming fumonisins (d18:0-P, as discussed above) [144]; and a mutation in an SPT subunit (Sptssb) that increases utilization of stearoyl-CoA and, thus, elevation of sphingolipids with d20-sphingoid bases (including d20:1-P) [34].

A recent review has discussed how different sphingoid base 1-P affect membrane structure and biological (and pathobiological) processes [142], such as the following: activation of S1P receptors is influenced by the alkyl chain length and varies with receptor subtypes [145]; sphinganine 1-phosphate and S1P have opposite effects on TGF-β-induced Smad2/3 phosphorylation and up-regulation of collagen synthesis (S1P activates and sphinganine 1-phosphate inhibits) [146]; d20:1-P displays partial agonism at the S1P2 receptor [147]; and phytosphingosine 1-phosphate also activates some S1P receptors [148,149]. These differences clearly warrant more frequent inclusion of these structural variants in lipidomic analyses.

### 4.2. N-Acyl-Sphingoid Bases

As noted earlier, the term “ceramides” is often used to reflect all N-acyl-sphingoid bases, whereas the name without quotation marks specifies N-acyl-sphingosines (e.g., N-palmitoylsphingosine would be abbreviated as d18:1/C16:0, or by the LIPID MAPS nomenclature Cer 18:1(4E);1OH,3OH/16:0) [28]. Likewise, N-acyl-sphinganines (with backbone d18:0 or by LIPID MAPS nomenclature SPB 18:0;1OH,3OH) are termed dihydroceramides, DhCer (but sometimes designated with quotation marks if other saturated backbone sphingoid bases, such as chain length variants, are included); and N-acyl-sphingoid bases with a phytosphingosine backbone (SPB 18:0;1OH,3OH,4OH) are often referred to as “phytoceramides” even though they are more literally “4-hydroxy-dihydroceramides”. The fatty acyl chain is sometimes specified by name (for example, N-palmitoylsphingosine) or by a number (such as C16-ceramide), or using an explicit abbreviation (Cer 18:1(4E);1OH,3OH/16:0 according to the original LIPID MAPS nomenclature [28,150]). In mammals, the fatty acyl chains of “ceramides” have been found to range from 2 carbons (i.e., acetyl) [151] to more than 36 carbon atoms, although most mammalian tissues have sphingolipids with chain lengths of 14 to 26 carbons (noteworthy exceptions being skin and testis, which have longer chain lengths) [71,152]. The fatty acids are primarily saturated and monounsaturated, but there are many instances where two or more double bonds are present (ceramides in testis, for example, have very-long-chain polyunsaturated fatty acids) [152]. Another feature of the fatty acids of mammalian “ceramides” is that they sometimes have an α- or ω-hydroxyl group. The latter is specifically found in skin ceramides [71], where it is often esterified to other long-chain fatty acids, especially linoleic acid, to form so-called acylceramides, which stabilize the lipid lamellae as well as become covalently attached to cornified envelope proteins [153]. There are so many varieties of skin “ceramides” that another series of abbreviations is often used in describing them [71,153].

N-acyl-sphingoid bases are extensively involved in cell regulation as the backbones of most of the complex sphingolipids of cells, skin, lipoproteins, and other biological structures and as dynamic players in membrane reorganization and second-messenger signaling. Thus, much has been written about their biosynthesis [154], trafficking [155], and cellular functions [156]. The major subspecies are analyzed in most lipidomic analyses, and the less frequently analyzed compounds are derivatives of the sphingoid bases already mentioned (see above), compounds with less common fatty acyls, and other types of derivatives that will be discussed in subsequent sections.

### 4.3. Lyso-Sphingolipids

**Figure 6:**
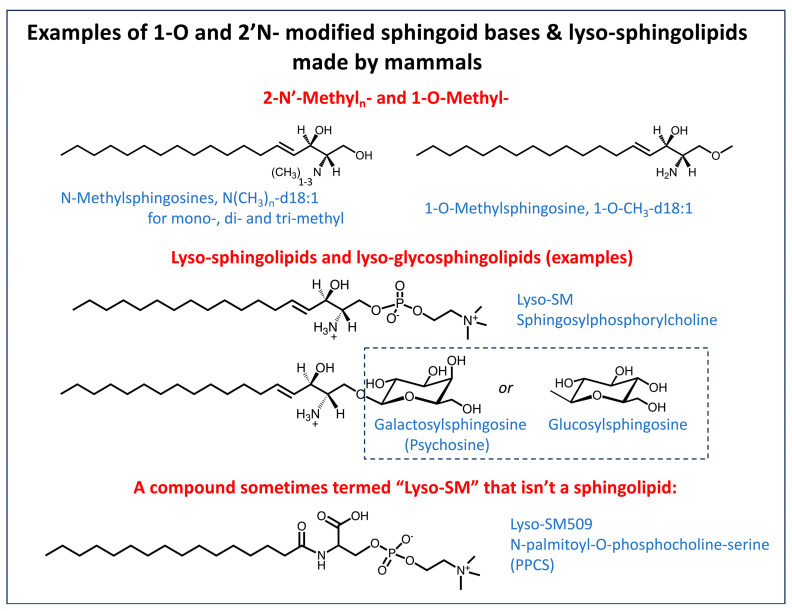
Examples of 1-O and 2’N- modified sphingoid bases and lyso-sphingolipids made by mammals. More information about these sphingoid base derivatives is provided in the text. One compound (Lyso-SM509) was found in a biomarker screen and was initially thought to be a lyso-SM, but the shown structure was deduced by later analyses, as explained in the text.

Sphingolipids that are missing the amide-linked fatty acyl but have a “headgroup” on the 1-hydroxyl are grouped under the subcategory of “lyso-” sphingolipids (Figure 6) [150]. They are variously named, sometimes by the specific structural components (the preferred naming from a lipidomics perspective), sometimes by a common name from a historical precedent, and often by affixing “lyso-” to the most closely related complex sphingolipid, for example, sphingosylphosphorylcholine (or lysosphingomyelin, lyso-SM) [157], glucosyl- and galactosyl-sphingosines (the latter is historically called psychosine), and de-acylated versions of more complex glycosphingolipids usually have the “lyso-” prefix [158,159]. Interest in these compounds most often arises from their notable appearance in plasma and/or tissue from sphingolipid storages diseases, and thus they are useful as biomarkers and sometimes in understanding the disease pathology. These diseases are as follows: Gaucher disease (glucosylsphingosine, lyso-GlcCer), Krabbe disease (galactosylsphingosine, lyso-GalCer), Gabry disease (globotriaosylsphingosine, lyso-Gb3), and Niemann–Pick disease (sphingosylphosphorylcholine, lyso-SM) [159,160]; GM1 gangliosidosis (lyso-monosialoganglioside GM1, lyso-GM1; lyso-asialoganglioside GM1, lyso-GA1), GM2 gangliosidosis (Tay Sachs, Sandhoff Disease) (lyso-monosialoganglioside GM2, lyso-GM2; lyso-asialoganglioside GM2, lyso-GA2), and metachromatic leukodystrophy (sulfogalactosylsphingosine, lyso-sulfatide) [158,159]; and others [159]. Some have been associated with signaling, particularly sphingosylphosphorylcholine (lyso-SM) and its receptor(s) that are at least partially shared with S1P [157,161,162], and the possibility that many of the lyso-sphingolipids may have functions beyond lipid storage diseases has been discussed, particularly in the context of cancer [159].

Relatively few studies of lyso-sphingolipids have reported data for backbone sphingoid bases other than sphingosine, despite knowing for a long time that the parent complex sphingolipids have more than one backbone, such as d18:0, d18:1, d18:2, t18:0, d20:0, t20:0, and d20:1 in sulfatides in human serum (interestingly, for these identifications the fatty acyl chains were removed from the complex sphingolipids by saponification prior to MS analysis) [43,163]. Studies of lyso-SM biomarkers of Niemann–Pick Type C disease type 1 discovered two subspecies using LC-MS/MS [164], one with the precursor/product ion pair for sphingosylphosphorylcholine (465 --> 184 *m*/*z*) and the other with 509 --> 184 *m*/*z* that was named “LysoSM-509” and speculated to be a Lyso-SM with an additional carbon dioxide. Both have also been seen in other studies of acid sphingomyelinase deficiency (Niemann–Pick type A/B) [165]. The structure of LysoSM-509 has been determined to not be a sphingolipid, but rather N-palmitoyl-O-phosphocholine-serine [166,167] (Figure 6), and plasma from Niemann–Pick Type C patients also had additional chain lengths (C14- to C24:1) [167].

It is likely that many of these lyso-sphingolipids and further structural variants will surface in untargeted lipidomics analyses; however, they will be particularly easy to miss because they are typically more water-soluble than most other sphingolipids and, in addition to being of low abundance, will be poorly extracted using many protocols.

### 4.4. N-Methylated Sphingoid Bases

Although rarely noted, mammals add methyl groups to the 1-hydroxyl- and 2-amino groups of sphingoid bases (Figure 2 and Figure 6). A 1-O-methyltransferase activity was reported for rat kidney and liver homogenates that utilizes S-adenosyl-L-methionine as the methyl donor [168]; in addition, 1-O-methylsphingosine can be produced as an artifact during the extraction and handling of sphingoid bases [169,170]. An N-methyltransferase activity (also using S-adenosyl-L-methionine) was found in mouse brain homogenates [171], and by radiolabeling studies using A431 cells in culture [172]. Little is known about these enzymes.

N-methylation of sphingoid bases in vivo was found in studies of the fate of Safingol (L-*threo*-sphinganine), a candidate anti-cancer drug, in TRAMP (transgenic adenocarcinoma of mouse prostate) mice [173]. After administration to the animals, it underwent extensive N-acylation, as assessed by LC-MS/MS analysis, but a portion of the L-*threo*-sphinganine appeared as mono-, di-, and tri-methyl-derivatives. The brain and lung had the mono-methyl-derivative in the highest proportion, whereas the liver had mostly the tri-methyl-amine [173]. The analyses also detected the methylation products of endogenous sphinganine and sphingosine, but they were not mentioned when we published this study because the amounts were very low [173]. Another sphingoid base analog (Enigmol [2S,3S,5S]-2-amino-3,5-dihydroxyoctadecane) has also been found to be methylated by HT29 cells to primarily the N,N,N-trimethyl-amino-derivative [174].

In contrast to the scarcity of studies of these compounds as metabolites, there have been many studies of the effects of exogenously adding N,N-dimethylsphingosine (DMS) to cells, in part because it has numerous biological activities, such as inhibition of protein kinase C [175] and sphingosine kinase [176,177]. Interest in endogenous N-methyl-sphingoid bases is growing, however, because they are beginning to surface in untargeted lipidomics analyses. In the first, an untargeted lipidomic analysis of Sprague–Dawley rats suffering from tibial-nerve transection as a model for neuropathic pain [178] found elevated DMS and suggested that it might be a pain mediator because injecting DMS intrathecally into healthy rats induced the development of mechanical allodynia in the hind paw. A follow-up study [179] examined whether DMS could be detected by LC MS/MS in patient samples from a Multiple Sclerosis Tissue Bank and found a greater abundance of ions with the appropriate characteristics for DMS in regions with disease lesions versus normal appearing white matter from the same patient. DMS was also detected in a human oligodendrocyte cell line (MO3.13) and was elevated by cuprizone, an inflammatory stimulus that mimics conditions associated with demyelination in multiple sclerosis. Several mechanisms have been proposed for this association between DMS and pain, and multiple subcategories of sphingolipids could be involved [180], including 1-deoxysphingoid bases, which are elevated in docetaxel chemotherapy [93].

It is not clear why the other methylated sphingoid bases (N-methyl- and N,N,N-tri-methyl-) were not noted in these studies; however, their ions might have been missed because they are not included in most data analysis programs. Furthermore, the major (and essentially only) cleavage product from positive ionization MS/MS of N,N,N-trimethylsphinganine is the trimethylammonium ion (*m*/*z* 60.1) [173], which is shared with many phospholipids. Another provocative possibility is that there might be additional metabolism of the N-methyl-sphingoid bases. In preliminary studies (unpublished), we examined the fate of N-methylsphingosine when added to cells in culture (HAP1 cells with a knockout of SPT, Horizon Discovery, https://horizondiscovery.com/, catalog ID HZGHC003579c012, URL accessed on 12 December 2024) and found, to our surprise, that it appeared in substantial amounts as N-methyl-ceramides with primarily C14-, C16-, and C-18-fatty acids, but not in downstream SM or glycosphingolipids. The possibility of formation of N-methyl derivatives of more complex sphingolipids cannot be ruled out, however, because N-methylated Gb3 has been noted in urine from Fabry disease patients [181].

Little is known about N-methylceramides, but an analysis of their effects on the multilamellar skin lipid model [100] reported that N-methyl-Cer decreased lipid chain order led to phase separation, improved cholesterol miscibility in the lipid membranes, and increased transmembrane water loss and the permeability to theophylline and indomethacin. There are many gaps in our knowledge about the occurrence and possible functions of this subcategory of sphingoid base metabolites.

## 5. Sphingoid Base Metabolites (Derivatives of More than One Functional Group—Mostly Metabolites of Ceramides)

The majority of the sphingolipids in most biological materials are complex sphingolipids with a “ceramide” backbone (Figure 1). As in Section 4, this section will provide an overview of the various subcategories, but the number of variations in the lipid backbones and headgroups of sphingolipids is too vast to discuss every component. Most of the sphingoid bases discussed in Section 3 should be kept in mind as potential backbones of these more complex sphingolipids.

### 5.1. Complex Phosphosphingolipids

Sphingomyelins (SM) are analyzed in almost all lipidomic studies of mammals. The first question to ask when examining sphingolipidomic data for SM is whether a method has been used that specifies the backbone and N-acyl components [182] or only the summed composition of the lipid backbone (i.e., the total number of carbons, hydrogens, etc. of the sphingoid base and fatty acyl). In positive ionization mode, the major product ion from collision-induced dissociation of SM is from the headgroup (*m*/*z* 184) [183]; therefore, much lipidomic data report the composition of the lipid backbone (i.e., sum of carbons, hydrogens, oxygens—possibly number of double bonds). If a full structural analysis has been conducted by LC-MS/MS or other methodology, the N-palmitoyl-SM shown in Figure 1 could be designated “SM·[1]18:1(4E);3OH/16:0” using the LIPID MAPS recommendations [28]; if not, then it might only be possible to designate it as SM 34:1;O2. Analysis of Cer1P is more informative because the LC elution position and backbone fragmentation products usually provide more information about the substituents [184].

Ceramide phosphoethanolanines (CerPE) comprise another subcategory of complex phosphosphingolipid in mammals, although they are usually found in only very small amounts (in contrast to insects and other organisms where they can be the major phosphosphingolipid) [185]. A recent MS imaging study reported that a CerPE with the composition 36:1;O2 was restricted within rosettes of a brain organoid tissue and suggested that it might be important for neuroprogenitor biology [186]. CerPE is biosynthesized by a member of the sphingomyelin synthase family, SMSr, that produces CerPE in the lumen of the ER [187]. SMSr is strongly expressed in the brain, testis, kidney, and pancreas and is detectable in all tissues [188], but its function is somewhat mysterious since so little CerPE is detected. It has been proposed that it serves a role in monitoring the levels of ceramide in the ER to prevent its buildup and induction of cell death [189]. A recent cryo-EM study of the CerPE synthesis activity found that it involves two processes—a phosphatidylethanolamine–phospholipase C hydrolysis, followed by subsequent transfer of the phosphoethanolamine moiety to ceramide [190]. It has been suggested that in addition to its CerPE synthesis activity, it can also act as a phosphatidylcholine–phospholipase C (PLC) to generate saturated fatty acid/monosaturated fatty acid-containing and docosahexaenoic acid (C22:6)-containing diacylglycerol species in cell signaling [191]

Ceramide 1-phosphates are becoming more regularly examined in lipidomic analyses since they are involved in numerous cell regulatory processes (cell proliferation, migration, inflammation, various cancers, and others) [192]. They are made by phosphorylation of ceramide by ceramide kinase or diacylglycerol kinase ζ [193].

An additional type of phosphoceramide is produced when SMs or CerPEs are cleaved to ceramide 1,3-cyclic phosphates by a category of phospholipase D found in the venom of brown recluse spiders and related spiders [194]. Similar enzymes have been found in bacteria and fungi, including components of the human GI microbiome; therefore, these lipids might be present in more “normal” circumstances and diseases than currently appreciated. The mechanism(s) of action of ceramide 1,3-cyclic phosphates are not known; however, it is interesting that they appear to not undergo hydrolysis by mammalian cells [195]. Furthermore, a chemically synthesized phytosphingosine-1,3-cyclic-phosphate (usually referred to as O-cyclic phytosphingosine-1-phosphate) has been used as an S1P mimetic and has multiple signaling and regulatory effects [196,197].

Ceramide phosphoinositols (PI-Cer, also called inositol phosphorylceramides, IPC) have been reported to be present in mouse [198] and human [199] brains; however, the reports did not provide definitive structural characterization and this category of sphingolipid is not known to be produced by mammals. They are made by fungi [139,200], plants [130], and protozoa [201,202,203] and are often elaborated to more complex glycans [130,200,204] termed glycosylinositol phosphoceramides (GIPC). Since mammals do not have the enzyme that makes IPC (IPC synthase), it is regarded to be a promising drug target for pathogenic yeast [205] and *Leishmania* [206]. It would, of course, be very interesting if these ions have been correctly identified as PI-Cer; however, they are more likely due to another isomeric or isobaric lipid.

### 5.2. Glycosphingolipids

Glycosphingolipids comprise the most structurally diverse and complex subcategory of mammalian sphingolipids and play important roles in membrane structure and cell function [207]. The metabolic branch point where glycosphingolipid biosynthesis begins (#6 in Figure 2) has two arms, one producing GlcCer and the other GalCer, each of which undergo further metabolism to more complex glycosphingolipids that are divided into root structure subcategories (e.g., ganglio-, globo-, etc., for GlcCer and Gala- for GalCer as briefly summarized in Figure 2) [208]. Individual molecular species are in the hundreds to thousands, depending on whether just the most abundant glycan and the lipid backbones are considered [209], and both serve as “codes” that determine their functions in neurons [210] and in other contexts [211]).

Lipidomics analyses rarely attempt to analyze more than a fraction of the glycosphingolipids in mammalian samples, and for good discussions of the issues that are involved in more comprehensive analysis, see [212,213,214]. The few glycosphingolipids that are most often quantified in lipidomics are so-called “HexCer” and “Hex_2_Cer” (mono- and di-hexosyl-ceramides) rather than identified by the specific glycans, which in mammals are primarily β-GlcCer and β-GalCer for HexCer and Galβ1-4Glc-Cer (LacCer) and Galβ1-4Gal-Cer (also known as DiGalCer or galabiosylceramide Ga2) for Hex_2_Cer [215,216].

Definitive structural characterization of all of the individual molecules will be challenging since there are so many isomers [209], but impressive progress in technologies is being made [214]). This subject is too expansive for much coverage here; however, following the theme of this review that structures will be highlighted when they are likely to be surprising, the following list gives some instances worth keeping in mind.

#### 5.2.1. Glucosylceramides and Galactosylceramides (HexCers)

HexCers are fairly widely reported in lipidomic studies, but it is actually GlcCer and GalCer that are present and both are found in essentially all tissues, albeit in widely ranging proportions. For example, the percentages of GlcCer and GalCer of some tissues are as follows (respectively): small and large intestines (99%, 1%); spleen (98%, 2%); liver (90%, 10%); lung (70%, 30%); thymus (62%, 18%); kidney (58%, 42%); lymph nodes (15%, 85%); and brain (3%, 97%) [217]. In addition, although only the β-glycosidic linkage is made by mammals, small amounts of the α-anomers have also been detected in the mammalian intestine [218], presumably derived from intestinal microflora that can make that anomer [219], and α-glycosylCer-producing bacteria have been found in tumors [220]. There are likely to be other instances where these lipids appear in mammalian tissues, considering the growing evidence that small amounts of sphingolipids from the GI microbiota are taken up by the intestines and transported to other tissues, such as the liver (see Section 6).

As noted earlier, there are methods to separate GlcCer and GalCer isomers (for example, [184,221,222]) and even the α- and β-anomers [217], but they are not amiable to high-throughput lipidomics. Considering the importance of knowing these distinct subcategories, analyses that are described as “sphingolipidomic” should include at least some analyses where these have been measured.

#### 5.2.2. Glycosphingolipids with Alkali-Labile Groups

Due to the stability of the linkages between sphingoid bases and most of the attached moieties (fatty acyls in amide linkage, carbohydrates in glycosyl-bonds, etc.), many of the extraction/work-up protocols for sphingolipids have used mild base to cleave the acyl ester bonds of glycerolipids to achieve better chromatographic separations and/or more sensitive and accurate mass spectrometric analysis. However, it has been pointed out [223] that this has interfered with the analysis of a number of alkali-labile sphingolipids, such as the following:

(a) GalCer with 3-O-acetylceramide backbones [224,225,226], which were first found by higher migration on thin-layer chromatography than typical glycosphingolipids (hence, they were referred to as “fast-migrating compounds, FMCs”). FMCs account for 15–35% of total myelin glycosphingolipid content and include GalCer with acylation of the hydroxyl of the amide linked α-hydroxy-fatty acids [227] or on hydroxyls of the galactose residue (see ref. [226,228]). These have mostly been found in myelin and myelinated nerve fibers [224,225,226] and cerebrospinal fluid [228], and have been suggested to contribute to autoimmune diseases such as multiple sclerosis due to similarities to microbial lipids [229,230].
(b) GalCer with acetyl groups on the carbohydrate hydroxyls are also considered to be components of the “fast-migrating cerebrosides” that were discussed above [224,225,226]. These have been found to be as complex as penta- and hexa-acetyl-cerebrosides and have been noted to resemble esterified carbohydrates of lipopolysaccharide and glycolipids found in many bacteria and thus candidates for autoimmune inflammation in central nervous system [228]. Another type of fast migrating cerebroside has either 3,4 or 4,6 cyclic acetal linkages between a fatty aldehyde and GalCer (these are termed plasmalocerebrosides) [231] or psychosine (Gal-sphingosine) (these are termed plasmalopsychosines) [232]; however, these linkages are labile under acidic conditions and stable to alkali.
(c) Gangliosides are labile to both acid and base. The sialic acid moieties are released from gangliosides at acid pH; however, some sialic acids are also O-acetylated and hydrolyzed under alkaline conditions [223]. Examples of gangliosides that have been found in an O-acetylated form are: a GT1b from mouse brain with the external sialic acid of the disialosyl chain, O-acetylated at position 9; O-acetylated-GD1a and O-acetylated-GM3 in erythrocytes; O-acetylated-GQ1b in mouse brain; and O-acetylated-GD3 in fetal rat brain and human melanoma [223]. O-acetylated gangliosides are mainly expressed during embryonic development and in the central nervous system in healthy adults, but their abnormal appearance can also be used as markers of cancers of neuroectodermal origin and as targets for cancer immunotherapy [233]. Clinical trial results have been mixed but multiple approaches are being taken to utilize the growing understanding of the roles of gangliosides of all types in cancer progression, detection, and treatment [234]. The functions of O-acetylation in normal tissues are only beginning to emerge, but it has been noted that one consequence of this modification is that O-acetylated-sialic acid is partially resistant to sialidase activity [223], which plays important roles in ganglioside function in the nervous system [235].
(d) Additional subcategories of sphingolipids with alkali-labile groups will be described in Section 5.3 and Section 5.4.

#### 5.2.3. Gangliosides with Alternative Sialic Acids

Although the predominant sialic acid of gangliosides is N-acetyl-neuraminic acid, smaller amounts of two other sialic acids are also found, N-acetyl-9-O-acetyl-neuraminic acid (Neu5,9Ac2) (discussed above) and N-glycolyl-neuraminic acid (Neu5Gc), which is primarily found in tumor tissues but very low levels have been detected in healthy human tissues [236]. Humans have not been thought to be able to synthesize Neu5Gc, so it was thought to be entirely taken up from dietary sources, such as red meat [237]; however, there are indications that human cancer cells can biosynthesize Neu5Gc under hypoxic conditions [236]. Humans generate antibody responses against ingested Neu5Gc, and red meat-derived Neu5Gc has been reported to promote inflammation and progression of cancer [238].

Gangliosides sometimes have an inner ester linkage between the sialic acid carboxyl group and one of the hydroxyl groups in the same ganglioside molecule, yielding a ganglioside lactone. This is a spontaneous reaction that can occur during the workup of lipid extracts under mildly acidic conditions, but this reactivity has also been suggested to be relevant biologically due to acidification of plasma membrane microcompartments (and there might also be an enzyme that catalyzes lactonization) [223]. Some examples of ganglioside lactones are the human brain GD1b-lactone, which increases with aging [239], and GD2-lactone in neuroblastoma and glioma [240]. The formation of ganglioside lactones greatly changes the conformation of the ganglioside, in addition to reversibly eliminating the negative charge of sialic acid and making the ganglioside resistant to sialidase, which might be part of the biological rationale for their formation [223].

### 5.3. 1-O-Acylceramides

Ester-linked fatty acyls are also found as another subcategory of “headgroup” for ceramides when the sample preparation does not utilize alkali cleavage. 1-O-acylceramides were first found in human and mouse epidermis [241] and then in other types of samples such as HCT116 colorectal carcinoma cells and livers from C57BL/6 mice [242]. The compounds in skin were comprised of a wide array of backbone sphingoid bases (d18:1 as the major subspecies and minor amounts of d16-, d17-, d19-, and d20-sphingosines and d18:2) and fatty acids ranging from C14- to C32- (with 0 or 1 double bond) for both the amide-linked and the 1-O-acyl groups [243], and were estimated to be present in substantial amounts (nearly a third) compared to the corresponding unmodified parent ceramides in skin [241]. The 1-O-acylceramides in HCT116 cells and mouse livers were associated with lipid droplets and increased upon feeding high-fat diets [242]. 1-O-acylceramide appears to be produced by two enzymes: a lysosomal phospholipase A2 with ceramide transacylase activity (PLA2G15) [244] and diacylglycerol acyltransferase 2 (DGAT2) on lipid droplets [242]. Targeting of 1-O-acylceramide metabolism has been proposed as a promising area for drug development [245] since both routes have important implications for disease; for example, PLA2G15 is a primary target for cationic amphiphilic drugs that cause phospholipidosis [246], and DGATs play important roles in cancer [247] and lipid droplets in many processes [248]. These findings with 1-O-acylceramides make it likely that they are present in many mammalian tissues but have been interpreted to be ceramides because the 1-acyl-fatty acids were lost during the sample workup.

### 5.4. ω-O-Acylglucosylceramides (And ω-O-Acylceramides)

ω-O-Acylceramides and ω-O-acylglucosylceramides are major components of skin and have an ester-linked fatty acid attached to the ω-hydroxyl- of ceramides that are novel in two respects—they are biosynthesized with an ω-hydroxyfatty acid in amide-linkage to the sphingoid base and these fatty acids are exceptionally long (30 to 36 carbon atoms) [249,250]. Over half of the ester-linked fatty acid is linoleic acid [251]. These are important in maintaining the permeability barrier of skin, epidermal proliferation and differentiation, modulation of skin immunity, and dermatologic disease [71,250,251]. Topical application of ceramides is also being used in the treatment of skin disease [71].

### 5.5. Sphingolipids Bound by Proteins

Subspecies of essentially every lipid category have been found to be covalently attached to proteins (e.g., fatty acids, cholesterol, sphingolipids, and others) [252], but this is rarely addressed in lipidomic analyses. Two types of sphingolipids have been found to be conjugated to protein.

#### 5.5.1. Acylceramides

Acylceramides are covalently bound to corneocyte surface proteins and are essential for the permeability barrier of skin [249]. This is a very complex family of compounds that have considerable variation in both the sphingoid bases (having both sphingosine and 6-hydroxysphingosines with odd and even chain lengths from 12 to 22 carbons) and fatty acids [31]. Two types of attachments between the protein and the sphingolipid have been proposed, one where the ω-hydroxyl of acylceramides is attached to the protein by transglutaminase (which is inferred from this activity in vitro) [253] and another where the fatty acid that is linked to the ω-hydroxyl of acylceramides (which is usually linoleic acid) is converted into an epoxy–enone that is covalently bound to the cornified envelope proteins [153]. As far as we are aware, little has been done to explore whether these types of linkages are found in other tissues.

#### 5.5.2. Inositol Phosphorylceramide-Glycans

Inositol phosphorylceramide-glycans are used to anchor proteins on the membranes of yeast [254] and protozoa (*Trypanosoma cruzi*) [201]. In general, these have a core glycan composed of mannose(α1-2)mannose(α1-6)mannose(α1-4)glucosamine that is attached (α1-6) to the myo-inositol of inositol phosphorylceramide (IPC), and a linker (usually ethanolamine phosphate or 2-aminoethylphosphonate) that is attached to the glycan core via its phosphate (often at the third mannose) and to the protein via its amine [201,204,255,256]. IPC is not thought to be made by mammals [257] (indeed, its synthase is thought to be a potential target for antifungal drugs [258]), but several mass spectrometric analyses have reported the presence of IPC (referred to as Cer-PI or PI-Cer) in brain based on the detection of ions with the expected mass to charge ratio (*m*/*z*) for such compounds [198,199,259]. These are likely to be misidentifications since there are other sphingolipids with the same *m*/*z*, and a recent study of traumatic injury in rat brain also detected ions consistent with PI-Cer but noted that this identification could not be confirmed with either MALDI MS/MS or LC-MS/MS, and perhaps the compounds are actually HexCer [260]. It is imaginable, nonetheless, that mammals might have a way of making these lipids, or perhaps obtain them from the diet or organisms in the GI tract, so further studies are warranted to explain these puzzling observations.

#### 5.5.3. Sphingolipids Non-Covalently Bound by Proteins

Numerous subcategories of sphingolipids have been found to be non-covalently bound by proteins [261,262,263,264,265,266], and there are probably more to be discovered since a bifunctional sphingosine reagent yielded over 180 candidate sphingolipid-binding proteins, some of which were already known to interact with sphingolipids [262]. This should be kept in mind in expanding the scope of sphingolipidomic analyses, as well as checking whether some of the sphingolipid extraction protocols might cause protein-bound sphingolipids to precipitate with their binding partners and interfere with their analysis.

## 6. Some Conditions That Can Affect, Often Unexpectedly, Sphingolipid Composition and Amounts

**Figure 7:**
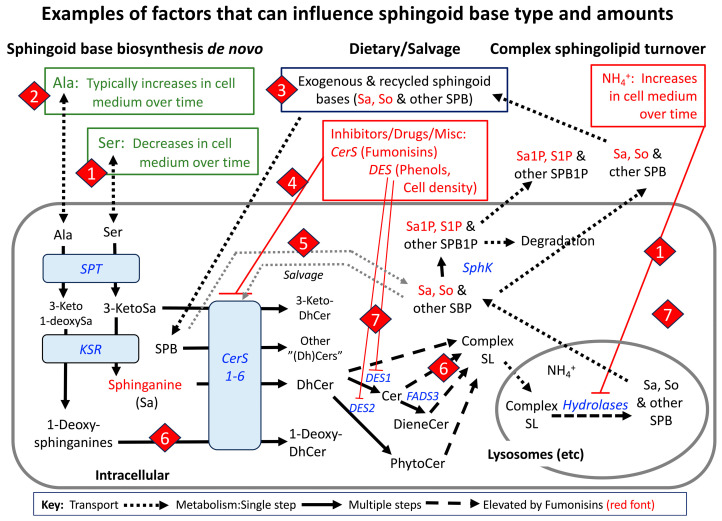
Examples of factors that can influence sphingoid base type and amount. This figure depicts sphingolipid biosynthesis and turnover in a manner analogous to Figure 2 (and the same abbreviations) but reorganized to display how several perturbations have been found in studies of sphingolipid metabolism, as discussed in Section 6.

This section describes some common occurrences and laboratory practices that can affect the amounts and types of sphingoid bases in cells in culture and sometimes in vivo as well as some emerging findings about the large numbers (and amounts) of sphingolipids that humans are exposed to in the diet and microbiome. An overall scheme depicting many of these is shown in Figure 7.

### 6.1. Changing the Medium of Cells in Culture Reveals Two Factors That Affect Sphingolipid Metabolism

In the early 1990s, we were surprised to find that whenever we routinely changed the medium of J774A.l cells in culture, there was a transient “burst” of sphingolipid metabolism that elevated by manyfold the amounts of free sphinganine, sphingosine, sphingoid base 1-phosphates, ceramides, and some other sphingolipids [267]. This phenomenon appears to be fairly common because elevations in sphingoid bases after changing the medium have also been observed for Swiss 3T3 cells [268] and NIH-3T3, A431, and NG108-15 cells [269] (other sphingolipids were not analyzed in that study).

In sorting through these observations using J774A.l cells, the increase in sphinganine was found to reflect de novo sphingoid base biosynthesis from Ser provided by the new medium [267] (Figure 7, diamond #1), which agreed with previous studies that had shown that the addition of extracellular serine above the ~millimolar Km of SPT increases de novo sphingoid base biosynthesis [270,271,272]. The increase in sphingosine, in contrast, was found [267] to arise mainly from the removal of ammonium ion that had accumulated to levels in the old medium (1 mM within hours to 3.4 mM by 36 h) that are sufficient to have lyso-osmotrophic effects on cells [273] and cause widespread suppression of sphingolipid turnover in acidic compartments [274] (Figure 7, diamond #2). Therefore, the sphingosine “burst” had brought to our attention something that others knew—that ammonium ion tends to accumulate in the medium over time (largely from glutamine turnover chemically and metabolically [273]—but had not been appreciated with respect to its consequences for sphingolipid metabolism—suppression of sphingolipid turnover until the ammonium ion is removed upon adding fresh medium [275].

It is noteworthy that the fates of the sphingoid bases were different [267]: most of the sphinganine burst (80–85%) was acylated and incorporated into (dihydro)ceramides and complex sphingolipids, whereas most (70%) of the sphingosine was phosphorylated to S1P and degraded, with much of the resulting ethanolamine phosphate appearing in phosphatidylethanolamine. Interestingly, if the acylation of sphinganine was blocked using the ceramide synthase inhibitor fumonisin B1, it was also diverted toward phosphorylation and degradation [267].

Part of the surprise in finding such a strong effect of adding Ser was that while many cells can biosynthesize that amino acid, it is utilized in many pathways [276,277]. Likewise, it was surprising to learn that the ammonium ion concentration of cells in culture can achieve levels that are lyso-osmotrophic; therefore, it can behave somewhat like drug-induced phospholipidosis [274]. The metabolites that were substantially affected by the “burst” included sphingoid bases, sphingoid base 1-phosphates, and other “bioactive sphingolipids” which might affect cell signaling. Indeed, there was evidence that the elevated sphingoid bases inhibited protein kinase C [278]. One wonders if there are conditions in vivo where high levels of ammonia impact sphingolipid metabolism in similar ways, such as in the placenta or tumors [273], or the GI tract from microbial metabolism [279].

### 6.2. Production of 1-Deoxy-Sphingoid Bases When Cell Culture Medium Is Depleted of Serine

Another consequence of changes in the concentration of serine and other amino acids in cell culture medium over time is the production of 1-deoxysphingolipids due to the utilization of Ala instead of Ser by serine palmitoyltransferase (Figure 7, diamond #3) (as discussed in Section 3.1.4). Although serine becomes depleted from the medium for many cell types, the concentration of alanine usually increases. For example, the Ser–Ala ratio at the beginning of cell culture is ~3:1 and, after 4 h, decreased to ~0.6:1 for J774A.1 cells and to ~0.5:1 for RAW264.7 cells [280]. For RAW264.7 cells, the ratio has elsewhere been reported [281] to decrease even lower after 3 and 4 days (to ~0.04:1 and ~0.01:1, respectively). We have measured the sphingolipid amounts of RAW264.7 cells at the beginning of culture and after 4 days and, as one would suspect, the ratio of ceramides to 1-deoxydihydroceramides (the major 1-deoxy-sphingolipid in the cells) decreased from ~3:1 at the beginning of cell culture to ~1:1 after 4 days in the medium [97]. This probably occurs more often than investigators realize because we have also noted increases in 1-deoxysphingolipids in LLC-PK1 and Vero cells maintained in culture for several days [81].

### 6.3. Elevation of Sphingoid Bases and 1-Deoxysphingoid Bases upon Ceramide Synthase Inhibition

Another perturbation of the sphingoid base composition of cells in culture and sometimes in vivo is the use of inhibitors to study sphingolipid metabolism and function. We first noticed this in uncovering that fumonisins inhibit ceramide synthase(s) (CerS) [282] (Figure 7, diamond #4). Fumonisins are mycotoxins that cause diseases in plants and animals that consume them, with damage to the liver, kidney, lung, brain, and other organs, altered immune function, developmental defects, and cancer (for a review, see [144]). Recent structural studies of CerS and fumonisins have characterized the inhibitory mechanism, including that fumonisins are N-acylated by CerS and bond tightly [283,284,285]. By inhibiting CerS so potently, they decrease the formation of more complex sphingolipids in all categories [286] and also cause accumulation of the substrates sphinganine and sphingosine. The elevation of sphinganine usually precedes sphingosine (since the latter arises from inhibition of salvage, diamond #5 in Figure 7) and is a useful biomarker for the early stages of exposure of animals to these mycotoxins [287]. Moreover, subsequent analyses discovered that in addition to these changes, cells in culture [144] and even humans exposed to fumonisins [288] show large increases in sphinganine 1-phosphate and sphingosine 1-phosphate (S1P) (via diamond #6, Figure 7), which implicates these bioactive metabolites in the abnormal cell signaling and disease caused by fumonisins [144,289,290]. Even more surprising, we noticed an unidentified sphingoid base accumulating in the cells and discovered that it is 1-deoxysphinganine, which had not been previously noticed in mammalian samples, presumably because 1-deoxysphingoid bases can be N-acylated by ceramide synthases [291] (Figure 7, diamond #7) and are difficult to detect in cells within the larger ceramide pool [81]. Another surprise from studies of the effects of fumonisins in vivo was that laboratory “chow” diets fed to rodents were sufficiently contaminated with fumonisins to affect the sphinganine biomarker [292], and subsequent analyses found fumonisins in agricultural feed and even commercial feline and canine food [293]. Thus, one should be mindful when using chow diets in studies of sphingolipid metabolism (and perhaps in feeding one’s pets).

All in all, CerS inhibition by fumonisins causes a “perfect storm” of perturbations in structural and signaling sphingolipids [144]. Many other naturally occurring inhibitors of CerS have been found and numerous synthetic compounds have been developed [294], but not all have been investigated as fully with respect to whether they also impact so many sphingolipid metabolites. One suspects that the paradigm that interference with one step of sphingolipid metabolism can impact the levels of multiple important structural and signaling sphingolipids is relevant to most or all inhibitors, sphingolipid analogs, and genetic alterations.

Although not an inhibitor of ceramide synthase *per se*, another inhibitor of ceramide production (myriocin, which inhibits serine palmitoyltransferase) is found in various medicinal preparations and extracts from *Isaria sinclairii* and *Cordyceps sinesis* that have been used in traditional Chinese medication and as nutritional supplements. A recent study in which extracts of commercially available *Cordyceps* were administered to obese mice at doses equivalent to human consumption found reduced ceramide and improvements in glucose homeostasis and other abnormalities [295]. If these findings lead to more extensive studies (and maybe the use of this readily available material in human studies), sphingolipidomic analyses of blood or other biological samples should prove very interesting.

### 6.4. Widespread Occurrence of Inhibitors of Dihydroceramide Desaturation, Causing Elevation of Dihydrosphingolipids

One of the interesting findings that surfaced early in studies of the enzymology of DhCer desaturase (DEGS1) was its inhibition by agents generally thought to be protective of enzymes, such as reductants (e.g., dithiothreitol) [296]. Then it surfaced that it is also inactivated by antioxidant phenols such as 4HPR (N-(4-hydroxyphenyl)retinamide or fenretinide) [112] and resveratrol [297], and a surprisingly wide range of additional compounds, including celecoxib, phenoxodiol, γ-tocopherol and γ-tocotrienol, 4HPA (N-(4-hydroxyphenyl)palmitamide), and AM404 (N-(4-hydroxyphenyl)arachidonamide or N-(4-hydroxyphenyl)-5Z,8Z,11Z,14Z-eicosatetraenamide)—a metabolite of acetaminophen with the potential to mediate some of its effects [298]. In addition to these compounds, some sphingosine kinase inhibitors [177] and S1P receptor antagonists [299] have been found to inhibit DEGS1. Phenolic compounds like most of the above can be envisioned to react directly with the catalytic center responsible for the desaturase reaction, and studies with 4HPR found that inhibition was initially competitive but became irreversible after longer incubation times [300]. A recent study found that many of the inhibitors (e.g., 4HPR, 4-HPA, AM404, phenoxodiol, and celesoxib) also induce polyubiquitination of DEGS1 and proteasomal degradation, which would contribute to, or possibly account for, the irreversible inhibition [298].

DEGS inhibition elevates DhCer (Figure 7, diamond #8), which have been assumed to be relatively innocuous from their inability to induce apoptosis when used at comparable concentrations to ceramides [301], however, their elevation due to DEGS1 inhibition by 4HPR was found to induce autophagy [112], which is another important cellular process. Over the last decade, dihydroceramides have been elevated from “Bit Players to Lead Actors” [52] as numerous studies have uncovered how they are both intermediates in the biosynthesis of ceramides and have important functions themselves and as the backbones of complex sphingolipids [52,53,297]. They have been placed at the center of major health problems in the U.S. and other nations—heart disease, cancer, type 2 diabetes, nonalcoholic fatty liver disease, West Nile Virus infection, and others, which also makes DEGS1 and (to some extent DEGS2) targets for pharmaceutical intervention [53,294,302,303].

Another surprising factor that has been found to reduce the desaturation of DhCer is cell density [304]. In studies of sphingolipids in neuroblastoma cells growing at low density versus those with density-induced growth arrest, the high-density cells had significantly elevated dihydroceramides and reduced ceramide, monohexosylceramide, and sphingomyelin. The elevated dihydroceramides were associated with lower DEGS activity, and various factors might account for this reduction, including levels of thiols in the medium and other factors that affect the redox state.

The breadth of factors that can affect DEGS1 activity is likely to have a widespread impact on the interpretation of sphingolipidomic analyses since DEGS1 inhibition is expected not only to elevate dihydroceramides but also the 14Z-isomer of sphingosine, since (as was discussed in Section 3.1.2 for the consequences of defective DEGS1 activity [63]), FADS3 is able to introduce a double bond directly into DhCer [56,62]. The (4E)- and (14Z)- isomers of sphingosine are probably not distinguished from each other by most currently used lipidomics protocols [63].

### 6.5. Conditions That Have Been Found to Affect the Composition of GlcCer and GalCer Under the Umbrella of “Monohexosylceramides”

As noted earlier, RAW264.7 cells incubated with (versus without) a TLR4 receptor agonist, Kdo_2_-Lipid A [140], displayed increases in GlcCer and GalCer of about the same fold (Figure 5). Nonetheless, transcriptomics analyses found that only the mRNA for UGCG was elevated after Kdo_2_-Lipid A stimulation, in agreement with the elevation of GlcCer, whereas the UGT8 mRNA was unchanged. On the other hand, a transcriptomic and sphingolipidomic analysis of human ovarian cancer tumors versus neighboring normal tissue [305] found that mRNA’s for GalCer synthase and sulfatide synthase (Gal3ST1) were elevated, and the predicted increases in GalCer and sulfatide were confirmed by LC-MS/MS analysis and tissue-imaging mass spectrometry. Hence, the elevation of GalCer appears to correlate sometimes with increased expression of UTG8 mRNA, but not always.

Mindful that GalCer is made in the lumen of the ER [13], perhaps their biosynthesis is limited by how rapidly (Dh)Cer is produced and exits the ER via CERT or vesicular trafficking versus “flipped” to the ER lumen where they are accessible to GalCer synthase. The rate of transbilayer transport might also be a factor, but Cer has been reported to cross phospholipid bilayers relatively rapidly [306,307]. The only investigations of this hypothesis that the author is aware of were conducted by Ying Liu as part of her doctoral thesis [308]. These studies used transfection of Hek293 cells with a dominant negative Sar1a mutant that is known to inhibit ER budding for trafficking to the Golgi [309]. Transfection elevated GalCer by 7-fold, with no change in mock-transfected cells (and no change in GlcCer) (Figure 4.5 of [308]). Studies using brefeldin A, which enables GalCer synthase to have longer access to (Dh)Cer by inducing Golgi fusion with the ER [310], also caused large increases in GalCer production (but not GlcCer) in Hek293, Hela, HepG2, and HL-60 cells (Figures 4.6 and 4.7 of [308]). These experiments indicate that processes that affect sphingolipid biosynthesis and trafficking from the ER influence the production of GalCer. If so, this might account for the changes in GalCer in the aformentioned experiments with RAW264.7 cells because Kdo_2_-Lipid A also induces autophagy [140], which involves de novo sphingolipid biosynthesis and alterations of ER behavior [311].

Another factor that now needs to be kept in mind is that UGT8 has recently been found to synthesize monogalactosyl diacylglycerol (MGDG) [312], a minor lipid of mammalian cells. Knockdown of UGT8 affected the unfolded protein response of the cells, which suggests that GalCer and/or MGDG might be involved in that important process.

These studies illustrate how differentiating between GlcCer and GalCer provides valuable information about this key branch point of glycosphingolipid biosynthesis.

### 6.6. Sphingolipids in Food and GI Microorganisms and Uptake from the GI Tract

Little is known about how many and how much of the sphingoid bases in the body arise from exogenous sources (Figure 7, diamond #3) versus from de novo biosynthesis. While all indications are that de novo biosynthesis is the major source, humans consume substantial amounts of sphingolipids [313,314] and are exposed to others from the many sphingolipid-producing microorganisms in the GI tract [51,137,315,316]. Estimations of the total amounts of sphingolipids that are consumed by humans have placed the amounts between 300–400 mg/day for the American diet [313] and 130–420 mg/day for low- and high-calorie prepared diets in Japan [314], which are in the same ballpark as cholesterol. It has been noted [317] that these are probably underestimates since there is little information regarding the glycosylinositol phosphoceramide (GIPC) content of plants and other organisms, and, for that matter, there have been no comprehensive, quantitative analyses of all of the sphingolipid subcategories in food. There are no estimations of the amounts that are taken up from GI microflora, however, reference [51] states that intestinal bacteria constitute approximately 1 g of sphingolipids in the gastrointestinal tract at any given time, and there is evidence that some uptake of sphingolipids from intestinal bacteria occurs (see below). Therefore, even a low percentage of uptake could have important consequences considering that, by even conservative estimates, humans consume over 100 g of sphingolipids every year and that is likely to be exceeded by the contributions from microorganisms in the GI tract.

Early studies on the fate of ingested sphingolipids (mostly those with sphingosine backbones) (summarized in [313]) found that phosphosphingolipids are largely hydrolyzed to “ceramides” and to some degree the backbone sphingoid bases in the small intestine by alkaline sphingomyelinase and neutral ceramidase [318]; whereas glycosphingolipids are cleaved less in the upper GI tract (involving lactase-phlorizin hydrolase with glycosylceramidase activity [319]) than the colon, where intestinal microflora are likely to participate (and a portion appears undigested in the feces). The sphingoid bases that are released are taken up by intestinal cells (and some intestinal bacteria [320]) and: (a) degraded and converted to fatty acids that appear in intestinal chylomicron triglycerides (which appear to account for most of the uptake); (b) incorporated into more complex sphingolipids that remain in the intestinal cells or secreted as ceramide in chyle [321]; or transported back out of the intestinal cells via P-glycoprotein [322,323]. These findings have been corroborated by studies of stable-isotope-labeled sphingosine uptake and metabolism by Caco-2/TC7 intestinal cells in culture [324]. These studies also monitored the N-acyl-metabolites (and sphingomyelins), which were the major metabolites in the cells, probably because the phosphorylated metabolite was primarily degraded (as has been seen earlier), and perhaps also because N-acylation played a role in the uptake/trapping of sphingosine by the cells [325]. It is noteworthy that essentially all of the intermediates and products of sphingolipid hydrolysis and resynthesis are bioactive compounds, so both the original complex sphingolipids in food and their metabolites have the potential to affect GI function, immune status, and microbiota [326].

The diversity of the sphingolipids in food is also fascinating because it includes essentially all of the backbone sphingoid bases shown in Figure 3 and Figure 4 and others [136,138,317,327,328] (even many 1-deoxy-sphingolipids [82,94,138]). A series of studies by Sugawara and associates (reviewed in [136]) have examined the absorption of many varieties of sphingolipids, including the subtypes shown in Figure 4 (plant, sea cucumber, and squid) and have found that some amounts of each appeared in lymph from cannulated rats, mostly as N-acyl-metabolites. Other studies [135] of the fate of similar sphingoid base types [the (4E, 8Z)- and (4E,8E)-plant sphingadienines, (4E,8E)-9-methyl-4,8-sphingadienine from mushrooms, and (4E,8E,10E)-9-methyl-4,8, 10-sphingatrienine from giant scallops] also found they were absorbed with small percentages appearing in lymph, ranging from ~1.2% for (4E,8Z)-sphingadienine to 0.1% for the (4E,8E,10E)-9-methyl-4,8,10-sphingatrienine. Differences in absorption appear to involve efflux of some subcategories of sphingoid bases (except sphingosine) from the intestinal by P-glycoprotein [322,323].

There are indications from dietary studies that some plant GlcCers can be taken up intact [136] and small amounts of sphingolipids novel structures (i.e., sphingoid base plus fatty acyl groups) have been found in lymph [329,330] to suggest that they have been absorbed intact rather than via the generally observed pathway—i.e., full digestion to sphingoid bases that are reacylated in intestinal cells before secretion. This is an intriguing possibility and warrants further study. To explore the fate of sphingolipids produced by GI microbes, a novel approach has been used [50,331]: to track the uptake and transfer of *Bacteroides* sphingolipids to the liver using sphingolipids made by the bacteria using palmitic acid alkyne, a sphingoid base precursor that allows the metabolites to be recovered using “click” chemistry. Interestingly, the sphingolipid that was transferred most from bacteria to host had a sphingoid base backbone derived from homoserine (Figure 4). Also, the studies found that homoserine-derived sphingolipids altered oxidative respiration and fat accumulation in HepG2 cells, a liver cell line.

Part of the interest in knowing if ingested sphingolipids are taken up intact and reutilized is that there have been several observations of sphingolipid consumption having benefits at sites distant from the GI tract, such as improvement of skin barrier functions in the epidermis [133,332,333]. However, it is not thought that the incorporation of the sphingolipids into the skin per se accounts for the benefits, but more likely via factors that affect sphingolipid biosynthesis by skin [136]. Dietary supplements with sphingolipids have been found in numerous studies to improve cognitive function [334,335] and to improve clinical symptoms of patients [336] with GM3 synthase deficiency as well as for GM3 synthase-deficient mice given diets enriched in milk gangliosides (GM3 and GD3) [337]. However, the latter study conducted an in-depth analysis of the sphingolipids in serum, liver, and brain and did not find any evidence for the presence of milk-derived GM3 or GD3 molecules after 3 months of feeding to wild-type mice and GM3 synthase-deficient mice. Therefore, while the mice studies support the hypothesis that GM3 and GD3 and/or their metabolites can affect brain development and improve symptoms of neurological disabilities, this appears to occur by other mechanism(s) than direct replacement of deficiencies in endogenous gangliosides by oral gangliosides. It is conceivable that a component of the sphingolipid supplement other than the signature sphingoid bases or carbohydrates (such as nervonic acid) might contribute to some of these responses [338]. However, many studies are uncovering beneficial effects of ingested sphingolipids due to modulation of GI microflora [136], including increases in the abundance of *Bifidobacterium* and decreases in *Bacteroidetes* [339], and reduced intestinal and systemic inflammation [317,340].

Immunomodulation by sphingolipids is an area of considerable interest since it might play a role in essentially all categories of chronic disease. It was initially uncovered by the finding of novel α-galactosylceramides from marine sponges that led to the development of KRN7000 as a potent CD1d ligand and activator of iNKT cells [341], and entered a new level of importance once it was appreciated that α-GalCer are also produced by human and mouse commensal bacteria [220]. Furthermore, CD1d binding and the nature of the iNKT cell response differ depending on the structures of the α-GalCer produced by different microorganisms [342]. The finding of microbial sphingolipids in tissues does not necessarily mean that they have been produced in the lumen of the GI tract, absorbed, and transported to the tissue because sphingolipid-producing bacteria have been found to infiltrate human tissues and tumors [220,343].

Before the immunomodulatory effects of sphingolipids were known, studies had shown that experimental animals fed diets that were supplemented with different categories of sphingolipids (ceramides, sphingomyelins, and glycosphingolipids in amounts similar to those found in food) developed fewer markers of early colon carcinogenesis and tumors than animals fed essentially sphingolipid-free diets [313,344]. The anti-cancer effects of ingested sphingolipids might involve the backbone sphingoid bases per se since a poorly metabolized analog (named Enigmol) was able to recapitulate the effects of feeding sphingolipids [174]. Subsequent studies have supported the potential for dietary sphingolipids to have chemopreventive and chemotherapeutic effects in colon cancer through multiple mechanisms of intestinal maintenance [317], including the new dimension that they might affect not only the intestinal cells but also the GI microflora [345]. Moreover, dietary glucosylceramides have been found to inhibit the growth of a head and neck squamous cell carcinoma xenograft in mice [346], which demonstrates that the benefits of dietary sphingolipids in cancer extend beyond the intestine.

Considering these recent findings, sphingolipidomic analyses of human tissues (and likewise other animals) should be mindful that bacterial sphingolipids might appear in tissues that harbor microorganisms that produce sphingolipids, or from GI microbes if they have been absorbed and transported to the liver and other tissues.

### 6.7. Sphingoid Base Degradative Metabolites and Chemical Artifacts

**Figure 8:**
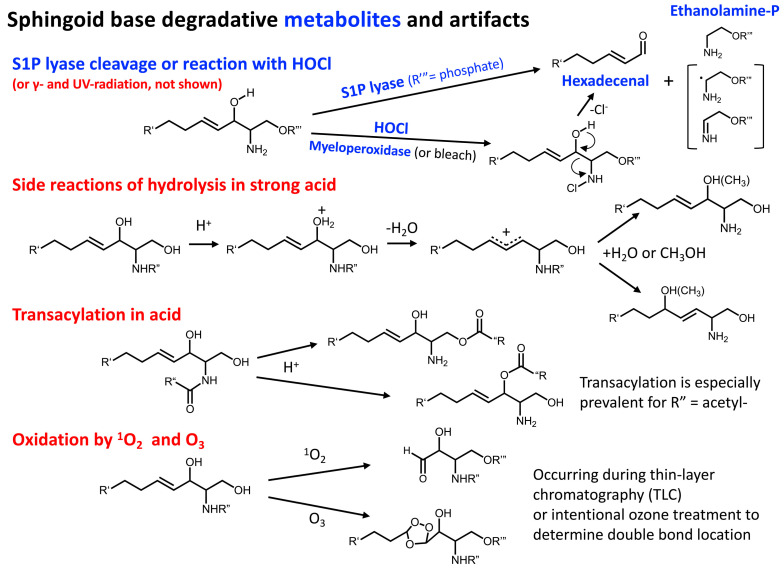
Sphingoid base degradative metabolites and artifacts. The upper enzyme-driven reactions produce the major turnover products from sphingoid bases; the other reactions and compounds have been reported to be produced during handling and common laboratory procedures, as described in the text.

Sphingoid bases undergo degradation via both enzymatic and chemical reactions that are summarized in this subsection and Figure 8.

#### 6.7.1. Sphingoid Base Degradative Metabolites

It warrants remembering that sphingolipid homeostasis is not a closed system, large amounts (how much does not appear to have been estimated) are lost by the two major pathways for elimination of sphingolipids from the body: (1) sloughing off from dead skin cells and excretion from the GI tract (including those consumed by GI microorganisms) (and likewise, loss from cells in culture to the medium in laboratory studies); and (2) enzymatic cleavage of sphingoid base 1-phosphates by S1P lyase (as depicted in Figure 2, diamond #10). Both should be borne in mind in interpreting research data since they represent disappearances that can complicate the interpretation of metabolic flux data, especially time course analyses. The products of the lyase reaction are ethanolamine phosphate and fatty aldehydes (whose structures depend on the nature of the sphingoid base), as represented by hexadecenal from S1P (Figure 8).

Fatty aldehydes can also be produced physiologically from an enzymatic reaction (production of hypochlorous acid, HOCl, by myeloperoxidase, a heme-containing enzyme that polymorphonuclear leukocytes use to kill bacteria [347]) followed by nonenzymatic decomposition of the chloramine intermediate [348]. This can also be produced by the treatment of sphingolipids with bleach and by free radical degradation of sphingoid bases in γ- and ultraviolet irradiation [348]. It appears that sphingolipid-derived fatty aldehydes serve regulatory functions in the cells (at low concentrations they enhance, but at higher concentrations inhibit, oxidative metabolism) [348]. Hexadecenal and other fatty aldehydes can be reutilized by reduction to fatty alcohols (which are incorporated into plasmalogens) [349] or oxidized to fatty acids [350], but they are also reactive species [351,352] that have been associated with the pathogenesis of Sjögren-Larsson syndrome, an inherited neurocutaneous disorder caused by mutations in the enzyme that catalyzes the oxidation of fatty aldehydes to fatty acids [353,354].

#### 6.7.2. Chemical Artifacts That Can Appear in Strong or Weak Acids (Figure 8)

Under strongly acidic hydrolysis conditions, it was first noted by Herb Carter and colleagues [169,170] that dehydration followed by rehydration (or addition of methanol if the reaction is conducted in that solvent) produce the original molecule, the 3-methyl-ether, and 5-hydroxy- (or methoxy-) 3-deoxysphingosine. In mild acid, acyl-chain migration can occur from the 2-amino-position to hydroxyls at positions 1 and 3, and this is especially prevalent for N-acetyl-sphingoid bases (C2-“ceramides”) [355] but also occurs with longer chain lengths (e.g., N-hexanoyl- and N-hexadecanoyl-). Furthermore, the 2N -> 1O and 2N -> 3O rearrangements occur with both N-acyl-sphinganines and -sphingosines, but the 4-*trans* double bond reduces 2N -> 3O migration due to the allylic nature of the 3OH. Chloroform and chloroform/methanol solvent mixtures were found to be sufficiently acidic to allow detectable rearrangement of N-acetyl-sphingosine solutions kept for long periods at −20 °C [355].

#### 6.7.3. Oxidative Damage to Sphingoid Bases (Figure 8)

As mentioned earlier, the double bond positions of sphingoid bases can be determined by MS after cleavage using ozone [64,85]. Just ambient air alone has been found to cause surface oxidation when sphingolipids are deposited on various surfaces, with the greatest occurring on silica gel thin-layer chromatography plates and the least on glass slides [356]. The oxidation was avoided by the protection of the sample from the air using N_2_ and by placing the samples in a dark room, indicating that light is involved in the singlet oxygen chemistry. Thus, the investigators warn that extra care is needed when lipid analyses involve surfaces where the unsaturated species can undergo oxidation.

In addition to these reactions, photo-oxidation of glycosphingolipids has been found to produce keto-, hydroxy-, and hydroperoxyl-derivatives of the unsaturated fatty acyl of the lipid backbone [357] and 8- and 9-hydroperoxides at the Δ8,9-double bond of the diene sphingoid base of plant GlcCer [358]. These have been referred to as “oxidized GlcCer” [358] and “oxidized ceramides” [359] and have also been reported to appear in tissues from mice given the per- and poly-fluoroalkyl substances (PFASs, also termed “forever chemicals”) that can perturb metabolic and reproductive health, but it is not evident that these are the same molecular species. There is clearly a need for additional studies of these types of compounds.

### 6.8. Other Types of Degradative Reactions, Chemical Artifacts and Analytical Complications

Sample handling before analysis is a potential source of many artifacts, such as errors in the estimation of sphingomyelin and ceramides due to rapid cleavage by phospholipase(s), overestimation of S1P in blood due to generation after drawing blood samples, etc. [360]. Degradation can occur from contaminants in extraction solvents (HCl and phosgene from chloroform, formaldehyde from methanol, peroxides from ethers, and others) (for more information, see the Avanti web talk by Robert Murphy: https://avantiresearch.com/news/tech-talks/solvent-challenges-associated-with-the-storing-and-extraction-of-lipids, accessed on 12 December 2024), and procedures that use acid or base in the sample work-up (as discussed above) [223]. We encountered another variable when a laboratory had difficulty reproducing our method for analysis of ceramide 1-phosphates [184] because some of the SM in extracts was undergoing hydrolysis unless extra care was taken in neutralizing the final step [361]. Not finding this ourselves, we eventually discovered that the degradation varied with the source of the borosilicate test tubes used for the extraction, presumably due to contaminants in the glass. Likewise, some but not all investigators have encountered difficulties in the analysis of Cer1P and S1P due to carryover and tailing from LC columns and have implemented solutions (see references [362,363,364] for example). Variable behaviors such as these are probably due to differences in the LC systems used (e.g., whether they provide metal surfaces that bind phosphates), sample sizes, solvents, salts, pH, flow rate, and other factors. Therefore, when the amounts of compounds change unexpectedly, or unprecedented sphingolipid structures are found, it is worthwhile to explore the possibility that there might be causes such as these before reporting the findings.

Further discussion of the technical and interpretive challenges of sphingolipidomic analysis is beyond the scope of this review, but can be found in recent publications that have: considered factors that can affect the analysis of sphingoid base and complex sphingolipids [365]; proposed recommendations for good practice in MS-based lipidomics [360] (as well as provided examples of pitfalls in MS analysis of mammalian samples [366]); called for the inclusion of key information about lipidomic experiments in publications to enhance the field’s consistency, comparability, and repeatability [367]; compared the results of ceramide analyses from multiple laboratories using a standard biologic material (human blood plasma from the National Institute of Standards and Technology) [368]; and provided an overview of the important issues in the selection of internal standards for accurate quantification of complex lipid species in biological extracts [369]. The latter issue is of tantamount importance for MS analysis of sphingolipids since they have such diverse structures and differences in ease of extraction, LC behavior, ionization, and fragmentation yields in MS and MS^n^ protocols—usually requiring different instrument settings for the backbone and chain-length variants versus an added internal standard (or standards) for many of the subcategories (for example, see [184]). Thus, quantitative comparisons should be examined carefully to ensure that the methodology has been validated for all of the sphingolipids that are being reported, which usually requires the addition of a wide range of internal standards and/or considerable experience with how structural variants compare to the limited number of internal standards that have been used in all aspects of the lipidomics analysis. Even then, the results can be impacted by changes in the samples to be analyzed (sample type and amount) and technical issues, such as age of the instrument, how recently it has been given periodic maintenance (which sometimes makes the performance with some sphingolipids worse!) and other factors. Fortunately, comparisons of fold differences between individual molecular subspecies of sphingolipids are impacted less by many of these factors, although they should nonetheless be kept in mind.

## 7. Concluding Comments and Perspectives

This is obviously not a complete list of sphingolipids that warrant further consideration in sphingolipidomic analyses, but it is hoped that these examples will encourage investigators to give more attention to individual subspecies, including less often analyzed compounds. The already-known sphingolipidome beckons the development of technologies capable of more comprehensive analysis, and there are many novel compounds to be added to it. When new compounds are identified, they should be entered into databases such as the LIPID MAPS^®^ Structure Database (https://www.lipidmaps.org/databases/lmsd/overview, accessed on 12 December 2024) and added to lipid analysis software. The lack of standards for most of these compounds, as well as for many of the sphingolipids that have been long known, is a technical impediment that should be addressed.

The field of sphingolipidology began with a search for the chemicals of life, and that is still worthwhile and necessary today. As stated by J. L. W. Thudichum in the preface to “A Treatise on the Chemical Constitution of the Brain” (1884) [370]:

“That all remedies…will have to be chemical agents is probably not subject to doubt…It is to reach this goal of complete knowledge that the medicinal chemist must spare no efforts, and it is to the increase of this knowledge that my best efforts during many years have been directed. The reader will thus be better able to appreciate the reasons which have caused me to give so much attention to the chemolysis of the principles extracted from the brain, and to surmise the grounds which influence me not to coincide with those who propose to avoid this laborious effort, and to carry on research by a kind of fishing for supposed disease-poisons, of which, according to my view of the subject, the attempt of the boy to catch a whale in his mother’s washing-tub is an appropriate parabole.”

## References

[1] Sullards M.C., Liu Y., Chen Y., Merrill A.H. (2011). Analysis of mammalian sphingolipids by liquid chromatography tandem mass spectrometry (LC-MS/MS) and tissue imaging mass spectrometry (TIMS). Biochim. Biophys. Acta.

[2] Peng B., Weintraub S.T., Coman C., Ponnaiyan S., Sharma R., Tews B., Winter D., Ahrends R. (2017). A Comprehensive High-Resolution Targeted Workflow for the Deep Profiling of Sphingolipids. Anal. Chem..

[3] Gao L., Ji S., Burla B., Wenk M.R., Torta F., Cazenave-Gassiot A. (2021). LICAR: An Application for Isotopic Correction of Targeted Lipidomic Data Acquired with Class-Based Chromatographic Separations Using Multiple Reaction Monitoring. Anal. Chem..

[4] Smirnov D., Mazin P., Osetrova M., Stekolshchikova E., Khrameeva E. (2021). The Hitchhiker’s Guide to Untargeted Lipidomics Analysis: Practical Guidelines. Metabolites.

[5] Chang J.K., Teo G., Pewzner-Jung Y., Cuthbertson D.J., Futerman A.H., Wenk M.R., Choi H., Torta F. (2023). Q-RAI data-independent acquisition for lipidomic quantitative profiling. Sci. Rep..

[6] Solosky A.M., Kirkwood-Donelson K.I., Odenkirk M.T., Baker E.S. (2024). Recent additions and access to a multidimensional lipidomic database containing liquid chromatography, ion mobility spectrometry, and tandem mass spectrometry information. Anal. Bioanal. Chem..

[7] Hartler J., Armando A.M., Trotzmuller M., Dennis E.A., Kofeler H.C., Quehenberger O. (2020). Automated Annotation of Sphingolipids Including Accurate Identification of Hydroxylation Sites Using MS(n) Data. Anal. Chem..

[8] Schindler R.L., Oloumi A., Tena J., Alvarez M.R.S., Liu Y., Grijaldo S., Barboza M., Jin L.W., Zivkovic A.M., Lebrilla C.B. (2024). Profiling Intact Glycosphingolipids with Automated Structural Annotation and Quantitation from Human Samples with Nanoflow Liquid Chromatography Mass Spectrometry. Anal. Chem..

[9] Peterka O., Maccelli A., Jirasko R., Vankova Z., Idkowiak J., Hrstka R., Wolrab D., Holcapek M. (2024). HILIC/MS quantitation of low-abundant phospholipids and sphingolipids in human plasma and serum: Dysregulation in pancreatic cancer. Anal. Chim. Acta.

[10] Dodds J.N., Baker E.S. (2019). Ion Mobility Spectrometry: Fundamental Concepts, Instrumentation, Applications, and the Road Ahead. J. Am. Soc. Mass Spectrom..

[11] Biricioiu M.R., Sarbu M., Ica R., Vukelic Z., Clemmer D.E., Zamfir A.D. (2024). Human Cerebellum Gangliosides: A Comprehensive Analysis by Ion Mobility Tandem Mass Spectrometry. J. Am. Soc. Mass Spectrom..

[12] Merrill A.H. (2011). Sphingolipid and glycosphingolipid metabolic pathways in the era of sphingolipidomics. Chem. Rev..

[13] Biran A., Santos T.C.B., Dingjan T., Futerman A.H. (2024). The Sphinx and the egg: Evolutionary enigmas of the (glyco)sphingolipid biosynthetic pathway. Biochim. Biophys. Acta Mol. Cell Biol. Lipids.

[14] Sandhoff R., Schulze H., Sandhoff K. (2018). Ganglioside Metabolism in Health and Disease. Prog. Mol. Biol. Transl. Sci..

[15] Harrison P.J., Dunn T.M., Campopiano D.J. (2018). Sphingolipid biosynthesis in man and microbes. Nat. Prod. Rep..

[16] Hanada K. (2024). Metabolic channeling of lipids via the contact zones between different organelles. BioEssays.

[17] Muralidharan S., Shimobayashi M., Ji S., Burla B., Hall M.N., Wenk M.R., Torta F. (2021). A reference map of sphingolipids in murine tissues. Cell Rep..

[18] Quinville B.M., Deschenes N.M., Ryckman A.E., Walia J.S. (2021). A Comprehensive Review: Sphingolipid Metabolism and Implications of Disruption in Sphingolipid Homeostasis. Int. J. Mol. Sci..

[19] Gable K., Gupta S.D., Han G., Niranjanakumari S., Harmon J.M., Dunn T.M. (2010). A disease-causing mutation in the active site of serine palmitoyltransferase causes catalytic promiscuity. J. Biol. Chem..

[20] Thudichum J.L.W. (1881). Researches on the chemical constitution of the non-phosphorized group of nitrogenous principles of the brain. Ann. Chem. Med..

[21] Klenk E. (1929). About Sphingosine. Notice about Cerebrosides. Z. Physiol. Chem..

[22] Carter H., Glick F.J., Norris W.P., Phillips G.E. (1947). Biochemistry of the sphingolipids. III. Structure of sphingosine. J. Biol. Chem..

[23] Carter H., Haines W.J., Ledyard W.E., Norris W.P. (1947). Biochemistry of the sphingolipids: I. Preparation of sphingolipids from beef brain and spinal cord. J. Biol. Chem..

[24] Pruett S.T., Bushnev A., Hagedorn K., Adiga M., Haynes C.A., Sullards M.C., Liotta D.C., Merrill A.H. (2008). Biodiversity of sphingoid bases (“sphingosines”) and related amino alcohols. J. Lipid Res..

[25] Carreira A.C., Santos T.C., Lone M.A., Zupancic E., Lloyd-Evans E., de Almeida R.F.M., Hornemann T., Silva L.C. (2019). Mammalian sphingoid bases: Biophysical, physiological and pathological properties. Prog. Lipid Res..

[26] Dingjan T., Futerman A.H. (2021). The role of the ’sphingoid motif’ in shaping the molecular interactions of sphingolipids in biomembranes. Biochim. Biophys. Acta Biomembr..

[27] Karlsson K.A. (1970). On the chemistry and occurrence of sphingolipid long-chain bases. Chem. Phys. Lipids.

[28] Liebisch G., Fahy E., Aoki J., Dennis E.A., Durand T., Ejsing C.S., Fedorova M., Feussner I., Griffiths W.J., Kofeler H. (2020). Update on LIPID MAPS classification, nomenclature, and shorthand notation for MS-derived lipid structures. J. Lipid Res..

[29] Liu N.J., Hou L.P., Bao J.J., Wang L.J., Chen X.Y. (2021). Sphingolipid metabolism, transport, and functions in plants: Recent progress and future perspectives. Plant Commun..

[30] Yumoto E., Sato M., Kubota T., Enomoto H., Miyamoto K., Yamane H., Koga J. (2021). Direct LC-ESI-MS/MS analysis of plant glucosylceramide and ceramide species with 8E and 8Z isomers of the long chain base. Biosci. Biotechnol. Biochem..

[31] Farwanah H., Pierstorff B., Schmelzer C.E., Raith K., Neubert R.H., Kolter T., Sandhoff K. (2007). Separation and mass spectrometric characterization of covalently bound skin ceramides using LC/APCI-MS and Nano-ESI-MS/MS. J. Chromatogr. B Anal. Technol. Biomed. Life Sci..

[32] Han G., Gupta S.D., Gable K., Niranjanakumari S., Moitra P., Eichler F., Brown R.H., Harmon J.M., Dunn T.M. (2009). Identification of small subunits of mammalian serine palmitoyltransferase that confer distinct acyl-CoA substrate specificities. Proc. Natl. Acad. Sci. USA.

[33] Hornemann T., Penno A., Rutti M.F., Ernst D., Kivrak-Pfiffner F., Rohrer L., von Eckardstein A. (2009). The SPTLC3 subunit of serine palmitoyltransferase generates short chain sphingoid bases. J. Biol. Chem..

[34] Zhao L., Spassieva S., Gable K., Gupta S.D., Shi L.Y., Wang J., Bielawski J., Hicks W.L., Krebs M.P., Naggert J. (2015). Elevation of 20-carbon long chain bases due to a mutation in serine palmitoyltransferase small subunit b results in neurodegeneration. Proc. Natl. Acad. Sci. USA.

[35] Russo S.B., Tidhar R., Futerman A.H., Cowart L.A. (2013). Myristate-derived d16:0 sphingolipids constitute a cardiac sphingolipid pool with distinct synthetic routes and functional properties. J. Biol. Chem..

[36] Kovilakath A., Mauro A.G., Valentine Y.A., Raucci F.J., Jamil M., Carter C., Thompson J., Chen Q., Beutner G., Yue Y. (2024). SPTLC3 Is Essential for Complex I Activity and Contributes to Ischemic Cardiomyopathy. Circulation.

[37] Glueck M., Koch A., Brunkhorst R., Ferreiros Bouzas N., Trautmann S., Schaefer L., Pfeilschifter W., Pfeilschifter J., Vutukuri R. (2022). The atypical sphingosine 1-phosphate variant, d16:1 S1P, mediates CTGF induction via S1P2 activation in renal cell carcinoma. FEBS J..

[38] Zeng J., Tan H., Huang B., Zhou Q., Ke Q., Dai Y., Tang J., Xu B., Feng J., Yu L. (2022). Lipid metabolism characterization in gastric cancer identifies signatures to predict prognostic and therapeutic responses. Front. Genet..

[39] Quehenberger O., Armando A.M., Brown A.H., Milne S.B., Myers D.S., Merrill A.H., Bandyopadhyay S., Jones K.N., Kelly S., Shaner R.L. (2010). Lipidomics reveals a remarkable diversity of lipids in human plasma. J. Lipid Res..

[40] Wertz P.W., Downing D.T. (1989). Free sphingosines in porcine epidermis. Biochim. Biophys. Acta.

[41] Sibille E., Berdeaux O., Martine L., Bron A.M., Creuzot-Garcher C.P., He Z., Thuret G., Bretillon L., Masson E.A. (2016). Ganglioside Profiling of the Human Retina: Comparison with Other Ocular Structures, Brain and Plasma Reveals Tissue Specificities. PLoS ONE.

[42] Merrill A.H., Wang E., Wertz P.W. (1986). Differences in the long chain (sphingoid) base composition of sphingomyelin from rats bearing Morris hepatoma 7777. Lipids.

[43] Taketomi T., Hara A., Uemura K., Kurahashi H., Sugiyama E. (1997). Preparation of various lysogangliosides including lyso-fucosyl GM1 and delayed extraction matrix-assisted laser desorption ionization time-of-flight mass spectrometric analysis. J. Biochem..

[44] Sonnino S., Chigorno V. (2000). Ganglioside molecular species containing C18- and C20-sphingosine in mammalian nervous tissues and neuronal cell cultures. Biochim. Biophys. Acta.

[45] Weishaupt N., Caughlin S., Yeung K.K., Whitehead S.N. (2015). Differential Anatomical Expression of Ganglioside GM1 Species Containing d18:1 or d20:1 Sphingosine Detected by MALDI Imaging Mass Spectrometry in Mature Rat Brain. Front. Neuroanat..

[46] Colsch B., Jackson S.N., Dutta S., Woods A.S. (2011). Molecular Microscopy of Brain Gangliosides: Illustrating their Distribution in Hippocampal Cell Layers. ACS Chem. Neurosci..

[47] Rosenberg A., Stern N. (1966). Changes in sphingosine and fatty acid components of the gangliosides in developing rat and human brain. J. Lipid Res..

[48] Ollen-Bittle N., Pejhan S., Pasternak S.H., Keene C.D., Zhang Q., Whitehead S.N. (2024). Co-registration of MALDI-MSI and histology demonstrates gangliosides co-localize with amyloid beta plaques in Alzheimer’s disease. Acta Neuropathol..

[49] Lone M.A., Hulsmeier A.J., Saied E.M., Karsai G., Arenz C., von Eckardstein A., Hornemann T. (2020). Subunit composition of the mammalian serine-palmitoyltransferase defines the spectrum of straight and methyl-branched long-chain bases. Proc. Natl. Acad. Sci. USA.

[50] Le H.H., Lee M.T., Besler K.R., Johnson E.L. (2022). Host hepatic metabolism is modulated by gut microbiota-derived sphingolipids. Cell Host Microbe.

[51] Bai X., Ya R., Tang X., Cai M. (2023). Role and interaction of bacterial sphingolipids in human health. Front. Microbiol..

[52] Siddique M.M., Li Y., Chaurasia B., Kaddai V.A., Summers S.A. (2015). Dihydroceramides: From Bit Players to Lead Actors. J. Biol. Chem..

[53] Magaye R.R., Savira F., Hua Y., Kelly D.J., Reid C., Flynn B., Liew D., Wang B.H. (2019). The role of dihydrosphingolipids in disease. Cell. Mol. Life Sci..

[54] Tzou F.Y., Hornemann T., Yeh J.Y., Huang S.Y. (2023). The pathophysiological role of dihydroceramide desaturase in the nervous system. Prog. Lipid Res..

[55] Grosch S., Schiffmann S., Geisslinger G. (2012). Chain length-specific properties of ceramides. Prog. Lipid Res..

[56] Jojima K., Kihara A. (2023). Metabolism of sphingadiene and characterization of the sphingadiene-producing enzyme FADS3. Biochim. Biophys. Acta Mol. Cell Biol. Lipids.

[57] Polito A.J., Akita T., Sweeley C.C. (1968). Gas chromatography and mass spectrometry of sphingolipid bases. Characterization of sphinga-4,14-dienine from plasma sphingomyelin. Biochemistry.

[58] Renkonen O., Hirvisalo E.L. (1969). Structure of plasma sphingadienine. J. Lipid Res..

[59] Baba T., Campbell J.L., Le Blanc J.C., Baker P.R. (2016). In-depth sphingomyelin characterization using electron impact excitation of ions from organics and mass spectrometry. J. Lipid Res..

[60] Zhao X., Wu G., Zhang W., Dong M., Xia Y. (2020). Resolving Modifications on Sphingoid Base and N-Acyl Chain of Sphingomyelin Lipids in Complex Lipid Extracts. Anal. Chem..

[61] Burla B., Oh J., Nowak A., Piraud N., Meyer E., Mei D., Bendt A.K., Studt J.D., Frey B.M., Torta F. (2024). Plasma and platelet lipidome changes in Fabry disease. Clin. Chim. Acta.

[62] Karsai G., Lone M., Kutalik Z., Brenna J.T., Li H., Pan D., von Eckardstein A., Hornemann T. (2020). FADS3 is a Delta14Z sphingoid base desaturase that contributes to gender differences in the human plasma sphingolipidome. J. Biol. Chem..

[63] Karsai G., Kraft F., Haag N., Korenke G.C., Hanisch B., Othman A., Suriyanarayanan S., Steiner R., Knopp C., Mull M. (2019). DEGS1-associated aberrant sphingolipid metabolism impairs nervous system function in humans. J. Clin. Investig..

[64] Steiner R., Saied E.M., Othman A., Arenz C., Maccarone A.T., Poad B.L., Blanksby S.J., von Eckardstein A., Hornemann T. (2016). Elucidating the chemical structure of native 1-deoxysphingosine. J. Lipid Res..

[65] Brydon S.C., Poad B.L.J., Fang M., Rustam Y.H., Young R.S.E., Mouradov D., Sieber O.M., Mitchell T.W., Reid G.E., Blanksby S.J. (2024). Cross-Validation of Lipid Structure Assignment Using Orthogonal Ion Activation Modalities on the Same Mass Spectrometer. J. Am. Soc. Mass Spectrom..

[66] Williams R.D., Wang E., Merrill A.H. (1984). Enzymology of long-chain base synthesis by liver: Characterization of serine palmitoyltransferase in rat liver microsomes. Arch. Biochem. Biophys..

[67] Gengatharan J.M., Handzlik M.K., Chih Z.Y., Ruchhoeft M.L., Secrest P., Ashley E.L., Green C.R., Wallace M., Gordts P., Metallo C.M. (2024). Altered sphingolipid biosynthetic flux and lipoprotein trafficking contribute to trans-fat-induced atherosclerosis. Cell Metab..

[68] Steele L., Drummond E., Nishida C., Yamamoto R., Branca F., Parsons Perez C., Allemandi L., Arnanz L., Schoj V., Khanchandani H.S. (2024). Ending Trans Fat-the First-ever Global Elimination Program for a Noncommunicable Disease Risk Factor: JACC International. J. Am. Coll. Cardiol..

[69] Omae F., Miyazaki M., Enomoto A., Suzuki M., Suzuki Y., Suzuki A. (2004). DES2 protein is responsible for phytoceramide biosynthesis in the mouse small intestine. Biochem. J..

[70] Mizutani Y., Kihara A., Igarashi Y. (2004). Identification of the human sphingolipid C4-hydroxylase, hDES2, and its up-regulation during keratinocyte differentiation. FEBS Lett..

[71] Uchida Y., Park K. (2021). Ceramides in Skin Health and Disease: An Update. Am. J. Clin. Dermatol..

[72] Watanabe T., Suzuki A., Ohira S., Go S., Ishizuka Y., Moriya T., Miyaji Y., Nakatsuka T., Hirata K., Nagai A. (2022). The Urinary Bladder is Rich in Glycosphingolipids Composed of Phytoceramides. J. Lipid Res..

[73] Dasgupta S., Kong J., Bieberich E. (2013). Phytoceramide in vertebrate tissues: One step chromatography separation for molecular characterization of ceramide species. PLoS ONE.

[74] Saigusa D., Shiba K., Inoue A., Hama K., Okutani M., Iida N., Saito M., Suzuki K., Kaneko T., Suzuki N. (2012). Simultaneous quantitation of sphingoid bases and their phosphates in biological samples by liquid chromatography/electrospray ionization tandem mass spectrometry. Anal. Bioanal. Chem..

[75] Kovacik A., Roh J., Vavrova K. (2014). The chemistry and biology of 6-hydroxyceramide, the youngest member of the human sphingolipid family. Chembiochem.

[76] Marques J.T., Cordeiro A.M., Viana A.S., Herrmann A., Marinho H.S., de Almeida R.F. (2015). Formation and Properties of Membrane-Ordered Domains by Phytoceramide: Role of Sphingoid Base Hydroxylation. Langmuir.

[77] Skolova B., Kovacik A., Tesar O., Opalka L., Vavrova K. (2017). Phytosphingosine, sphingosine and dihydrosphingosine ceramides in model skin lipid membranes: Permeability and biophysics. Biochim. Biophys. Acta Biomembr..

[78] Pullmannova P., Curikova-Kindlova B.A., Ondrejcekova V., Kovacik A., Dvorakova K., Dulanska L., Georgii R., Majcher A., Maixner J., Kucerka N. (2023). Polymorphism, Nanostructures, and Barrier Properties of Ceramide-Based Lipid Films. ACS Omega.

[79] Penno A., Reilly M.M., Houlden H., Laura M., Rentsch K., Niederkofler V., Stoeckli E.T., Nicholson G., Eichler F., Brown R.H. (2010). Hereditary sensory neuropathy type 1 is caused by the accumulation of two neurotoxic sphingolipids. J. Biol. Chem..

[80] Rotthier A., Auer-Grumbach M., Janssens K., Baets J., Penno A., Almeida-Souza L., Van Hoof K., Jacobs A., De Vriendt E., Schlotter-Weigel B. (2010). Mutations in the SPTLC2 subunit of serine palmitoyltransferase cause hereditary sensory and autonomic neuropathy type I. Am. J. Hum. Genet..

[81] Zitomer N.C., Mitchell T., Voss K.A., Bondy G.S., Pruett S.T., Garnier-Amblard E.C., Liebeskind L.S., Park H., Wang E., Sullards M.C. (2009). Ceramide synthase inhibition by fumonisin B1 causes accumulation of 1-deoxysphinganine: A novel category of bioactive 1-deoxysphingoid bases and 1-deoxydihydroceramides biosynthesized by mammalian cell lines and animals. J. Biol. Chem..

[82] Duan J., Merrill A.H. (2015). 1-Deoxysphingolipids Encountered Exogenously and Made de Novo: Dangerous Mysteries inside an Enigma. J. Biol. Chem..

[83] Lone M.A., Santos T., Alecu I., Silva L.C., Hornemann T. (2019). 1-Deoxysphingolipids. Biochim. Biophys. Acta Mol. Cell Biol. Lipids.

[84] Karsai G., Steiner R., Kaech A., Lone M.A., von Eckardstein A., Hornemann T. (2021). Metabolism of HSAN1- and T2DM-associated 1-deoxy-sphingolipids inhibits the migration of fibroblasts. J. Lipid Res..

[85] Poad B.L.J., Maccarone A.T., Yu H., Mitchell T.W., Saied E.M., Arenz C., Hornemann T., Bull J.N., Bieske E.J., Blanksby S.J. (2018). Differential-Mobility Spectrometry of 1-Deoxysphingosine Isomers: New Insights into the Gas Phase Structures of Ionized Lipids. Anal. Chem..

[86] Kirschbaum C., Saied E.M., Greis K., Mucha E., Gewinner S., Schollkopf W., Meijer G., von Helden G., Poad B.L.J., Blanksby S.J. (2020). Resolving Sphingolipid Isomers Using Cryogenic Infrared Spectroscopy. Angew. Chem. Int. Ed. Engl..

[87] Momin A.A., Park H., Allegood J.C., Leipelt M., Kelly S.L., Merrill A.H., Hanada K. (2009). Characterization of mutant serine palmitoyltransferase 1 in LY-B cells. Lipids.

[88] Lemieux R.U., Von Rudloff E. (1955). Periodate-permanganate oxidations. I. Oxidation of olefins. Can. J. Chem..

[89] Zuellig R.A., Hornemann T., Othman A., Hehl A.B., Bode H., Guntert T., Ogunshola O.O., Saponara E., Grabliauskaite K., Jang J.H. (2014). Deoxysphingolipids, novel biomarkers for type 2 diabetes, are cytotoxic for insulin-producing cells. Diabetes.

[90] Mwinyi J., Bostrom A., Fehrer I., Othman A., Waeber G., Marti-Soler H., Vollenweider P., Marques-Vidal P., Schioth H.B., von Eckardstein A. (2017). Correction: Plasma 1-deoxysphingolipids are early predictors of incident type 2 diabetes mellitus. PLoS ONE.

[91] Gorden D.L., Myers D.S., Ivanova P.T., Fahy E., Maurya M.R., Gupta S., Min J., Spann N.J., McDonald J.G., Kelly S.L. (2015). Biomarkers of NAFLD progression: A lipidomics approach to an epidemic. J. Lipid Res..

[92] Weyler J., Verrijken A., Hornemann T., Vonghia L., Dirinck E., von Eckardstein A., Vanwolleghem T., Michielsen P., Peiffer F., Driessen A. (2021). Association of 1-deoxy-sphingolipids with steatosis but not steatohepatitis nor fibrosis in non-alcoholic fatty liver disease. Acta Diabetol..

[93] Becker K.A., Uerschels A.K., Goins L., Doolen S., McQuerry K.J., Bielawski J., Sure U., Bieberich E., Taylor B.K., Gulbins E. (2020). Role of 1-Deoxysphingolipids in docetaxel neurotoxicity. J. Neurochem..

[94] Wan J., Li J., Bandyopadhyay S., Kelly S.L., Xiang Y., Zhang J., Merrill A.H., Duan J. (2019). Analysis of 1-Deoxysphingoid Bases and Their N-Acyl Metabolites and Exploration of Their Occurrence in Some Food Materials. J. Agric. Food Chem..

[95] Salcedo M., Cuevas C., Alonso J.L., Otero G., Faircloth G., Fernandez-Sousa J.M., Avila J., Wandosell F. (2007). The marine sphingolipid-derived compound ES 285 triggers an atypical cell death pathway. Apoptosis.

[96] Wang T., Wang Z., de Fabritus L., Tao J., Saied E.M., Lee H.J., Ramazanov B.R., Jackson B., Burkhardt D., Parker M. (2021). 1-deoxysphingolipids bind to COUP-TF to modulate lymphatic and cardiac cell development. Dev. Cell.

[97] Jimenez-Rojo N., Sot J., Busto J.V., Shaw W.A., Duan J., Merrill A.H., Alonso A., Goni F.M. (2014). Biophysical properties of novel 1-deoxy-(dihydro)ceramides occurring in mammalian cells. Biophys. J..

[98] Santos T.C.B., Vaz A., Ventura A.E., Saied E.M., Arenz C., Fedorov A., Prieto M., Silva L.C. (2020). Canonical and 1-Deoxy(methyl) Sphingoid Bases: Tackling the Effect of the Lipid Structure on Membrane Biophysical Properties. Langmuir.

[99] Santos T.C.B., Saied E.M., Arenz C., Fedorov A., Prieto M., Silva L.C. (2021). The long chain base unsaturation has a stronger impact on 1-deoxy(methyl)-sphingolipids biophysical properties than the structure of its C1 functional group. Biochim. Biophys. Acta Biomembr..

[100] Kovacik A., Pullmannova P., Pavlikova L., Maixner J., Vavrova K. (2020). Behavior of 1-Deoxy-, 3-Deoxy- and N-Methyl-Ceramides in Skin Barrier Lipid Models. Sci. Rep..

[101] Stoffel W., LeKim D., Sticht G. (1968). Metabolism of sphingosine bases. 8. Distribution, isolation and properties of D-3-oxosphinganine reductase. Stereospecificity of the NADPH-dependent reaction of 3-oxodihydrospingosine (2-amino-1-hydroxyoctadecane-3-one). Hoppe Seylers Z. Physiol. Chem..

[102] Braun P.E., Snell E.E. (1968). Biosynthesis of sphingolipid bases. II. Keto intermediates in synthesis of sphingosine and dihydrosphingosine by cell-free extracts of Hansenula ciferri. J. Biol. Chem..

[103] Morell P., Radin N.S. (1970). Specificity in ceramide biosynthesis from long chain bases and various fatty acyl coenzyme A’s by brain microsomes. J. Biol. Chem..

[104] Merrill A.H., Sullards M.C., Allegood J.C., Kelly S., Wang E. (2005). Sphingolipidomics: High-throughput, structure-specific, and quantitative analysis of sphingolipids by liquid chromatography tandem mass spectrometry. Methods.

[105] Beeler T., Bacikova D., Gable K., Hopkins L., Johnson C., Slife H., Dunn T. (1998). The Saccharomyces cerevisiae TSC10/YBR265w gene encoding 3-ketosphinganine reductase is identified in a screen for temperature-sensitive suppressors of the Ca^2+^-sensitive csg2Δ mutant. J. Biol. Chem..

[106] Kihara A., Igarashi Y. (2004). FVT-1 is a mammalian 3-ketodihydrosphingosine reductase with an active site that faces the cytosolic side of the endoplasmic reticulum membrane. J. Biol. Chem..

[107] Gupta S.D., Gable K., Han G., Borovitskaya A., Selby L., Dunn T.M., Harmon J.M. (2009). Tsc10p and FVT1: Topologically distinct short-chain reductases required for long-chain base synthesis in yeast and mammals. J. Lipid Res..

[108] Stankeviciute G., Tang P., Ashley B., Chamberlain J.D., Hansen M.E.B., Coleman A., D’Emilia R., Fu L., Mohan E.C., Nguyen H. (2022). Convergent evolution of bacterial ceramide synthesis. Nat. Chem. Biol..

[109] Uchendu C.G., Guan Z., Klein E.A. (2024). Spatial organization of bacterial sphingolipid synthesis enzymes. J. Biol. Chem..

[110] Ren J., Snider J., Airola M.V., Zhong A., Rana N.A., Obeid L.M., Hannun Y.A. (2018). Quantification of 3-ketodihydrosphingosine using HPLC-ESI-MS/MS to study SPT activity in yeast Saccharomyces cerevisiae. J. Lipid Res..

[111] Momin A.A. (2010). Application of Bioinformatics in Studies of Sphingolipid Biosynthesis.

[112] Zheng W., Kollmeyer J., Symolon H., Momin A., Munter E., Wang E., Kelly S., Allegood J.C., Liu Y., Peng Q. (2006). Ceramides and other bioactive sphingolipid backbones in health and disease: Lipidomic analysis, metabolism and roles in membrane structure, dynamics, signaling and autophagy. Biochim. Biophys. Acta.

[113] Ardail D., Popa I., Alcantara K., Pons A., Zanetta J.P., Louisot P., Thomas L., Portoukalian J. (2001). Occurrence of ceramides and neutral glycolipids with unusual long-chain base composition in purified rat liver mitochondria. FEBS Lett..

[114] Spassieva S.D., Markham J.E., Hille J. (2002). The plant disease resistance gene Asc-1 prevents disruption of sphingolipid metabolism during AAL-toxin-induced programmed cell death. Plant J..

[115] Shamshiddinova M., Gulyamov S., Kim H.J., Jung S.H., Baek D.J., Lee Y.M. (2021). A Dansyl-Modified Sphingosine Kinase Inhibitor DPF-543 Enhanced De Novo Ceramide Generation. Int. J. Mol. Sci..

[116] Govers S.K., Jacobs-Wagner C. (2020). Caulobacter crescentus: Model system extraordinaire. Curr. Biol..

[117] Pilz R., Opalka L., Majcher A., Grimm E., Van Maldergem L., Mihalceanu S., Schakel K., Enk A., Aubin F., Bursztejn A.C. (2022). Formation of keto-type ceramides in palmoplantar keratoderma based on biallelic KDSR mutations in patients. Hum. Mol. Genet..

[118] Boyden L.M., Vincent N.G., Zhou J., Hu R., Craiglow B.G., Bayliss S.J., Rosman I.S., Lucky A.W., Diaz L.A., Goldsmith L.A. (2017). Mutations in KDSR Cause Recessive Progressive Symmetric Erythrokeratoderma. Am. J. Hum. Genet..

[119] Takeichi T., Torrelo A., Lee J.Y.W., Ohno Y., Lozano M.L., Kihara A., Liu L., Yasuda Y., Ishikawa J., Murase T. (2017). Biallelic Mutations in KDSR Disrupt Ceramide Synthesis and Result in a Spectrum of Keratinization Disorders Associated with Thrombocytopenia. J. Investig. Dermatol..

[120] Bariana T.K., Labarque V., Heremans J., Thys C., De Reys M., Greene D., Jenkins B., Grassi L., Seyres D., Burden F. (2019). Sphingolipid dysregulation due to lack of functional KDSR impairs proplatelet formation causing thrombocytopenia. Haematologica.

[121] Wu L., Zhang Y., Zi J., Yan Y., Yu L., Lin D., Huang L., Lai X., Liao X., Yang L. (2022). Case report: Compound heterozygous mutations in the KDSR gene cause progressive keratodermia and thrombocytopenia. Front. Pediatr..

[122] Altawil L., Alshihry H., Alfaraidi H., Alhashem A., Alhumidi A., Alkuraya F.S. (2021). Progressive symmetrical erythrokeratoderma manifesting as harlequin-like ichthyosis with severe thrombocytopenia secondary to a homozygous 3-ketodihydrosphingosine reductase mutation. JAAD Case Rep..

[123] Park K.H., Ye Z.W., Zhang J., Hammad S.M., Townsend D.M., Rockey D.C., Kim S.H. (2019). 3-ketodihydrosphingosine reductase mutation induces steatosis and hepatic injury in zebrafish. Sci. Rep..

[124] Czuchlewski D.R., Csernus B., Bubman D., Hyjek E., Martin P., Chadburn A., Knowles D.M., Cesarman E. (2008). Expression of the follicular lymphoma variant translocation 1 gene in diffuse large B-cell lymphoma correlates with subtype and clinical outcome. Am. J. Clin. Pathol..

[125] Ordonez Y.F., Gonzalez J., Bedia C., Casas J., Abad J.L., Delgado A., Fabrias G. (2016). 3-Ketosphinganine provokes the accumulation of dihydroshingolipids and induces autophagy in cancer cells. Mol. Biosyst..

[126] Liu Q., Chan A.K.N., Chang W.H., Yang L., Pokharel S.P., Miyashita K., Mattson N., Xu X., Li M., Lu W. (2022). 3-Ketodihydrosphingosine reductase maintains ER homeostasis and unfolded protein response in leukemia. Leukemia.

[127] Spears M.E., Lee N., Hwang S., Park S.J., Carlisle A.E., Li R., Doshi M.B., Armando A.M., Gao J., Simin K. (2022). De novo sphingolipid biosynthesis necessitates detoxification in cancer cells. Cell Rep..

[128] Momin A.A., Park H., Portz B.J., Haynes C.A., Shaner R.L., Kelly S.L., Jordan I.K., Merrill J.A.H. (2011). A method for visualization of “omic” datasets for sphingolipid metabolism to predict potentially interesting differences. J. Lipid Res..

[129] Shankavaram U.T., Reinhold W.C., Nishizuka S., Major S., Morita D., Chary K.K., Reimers M.A., Scherf U., Kahn A., Dolginow D. (2007). Transcript and protein expression profiles of the NCI-60 cancer cell panel: An integromic microarray study. Mol. Cancer Ther..

[130] Cassim A.M., Grison M., Ito Y., Simon-Plas F., Mongrand S., Boutte Y. (2020). Sphingolipids in plants: A guidebook on their function in membrane architecture, cellular processes, and environmental or developmental responses. FEBS Lett..

[131] Fernandes C.M., Goldman G.H., Del Poeta M. (2018). Biological Roles Played by Sphingolipids in Dimorphic and Filamentous Fungi. mBio.

[132] Usmani S.A., Kumar M., Arya K., Ali B., Bhardwaj N., Gaur N.A., Prasad R., Singh A. (2023). Beyond membrane components: Uncovering the intriguing world of fungal sphingolipid synthesis and regulation. Res. Microbiol..

[133] Duan J., Ishida M., Aida K., Tsuduki T., Zhang J., Manabe Y., Hirata T., Sugawara T. (2016). Dietary Cerebroside from Sea Cucumber (Stichopus japonicus): Absorption and Effects on Skin Barrier and Cecal Short-Chain Fatty Acids. J. Agric. Food Chem..

[134] Ryan E., Gonzalez Pastor B., Gethings L.A., Clarke D.J., Joyce S.A. (2023). Lipidomic Analysis Reveals Differences in Bacteroides Species Driven Largely by Plasmalogens, Glycerophosphoinositols and Certain Sphingolipids. Metabolites.

[135] Mikami D., Sakai S., Nishimukai M., Yuyama K., Mukai K., Igarashi Y. (2021). Structure-dependent absorption of atypical sphingoid long-chain bases from digestive tract into lymph. Lipids Health Dis..

[136] Sugawara T. (2022). Sphingolipids as Functional Food Components: Benefits in Skin Improvement and Disease Prevention. J. Agric. Food Chem..

[137] Ryan E., Joyce S.A., Clarke D.J. (2023). Membrane lipids from gut microbiome-associated bacteria as structural and signalling molecules. Microbiology.

[138] Hu Z., Duan J. (2022). 1-Deoxysphingolipids and Their Analogs in Foods: The Occurrence and Potential Impact on Human Health. J. Nutr. Sci. Vitaminol..

[139] Mota Fernandes C., Del Poeta M. (2020). Fungal sphingolipids: Role in the regulation of virulence and potential as targets for future antifungal therapies. Expert Rev. Anti-Infect. Ther..

[140] Sims K., Haynes C.A., Kelly S., Allegood J.C., Wang E., Momin A., Leipelt M., Reichart D., Glass C.K., Sullards M.C. (2010). Kdo2-lipid A, a TLR4-specific agonist, induces de novo sphingolipid biosynthesis in RAW264.7 macrophages, which is essential for induction of autophagy. J. Biol. Chem..

[141] Burla B., Arita M., Arita M., Bendt A.K., Cazenave-Gassiot A., Dennis E.A., Ekroos K., Han X., Ikeda K., Liebisch G. (2018). MS-based lipidomics of human blood plasma: A community-initiated position paper to develop accepted guidelines. J. Lipid Res..

[142] Glueck M., Lucaciu A., Subburayalu J., Kestner R.I., Pfeilschifter W., Vutukuri R., Pfeilschifter J. (2024). Atypical sphingosine-1-phosphate metabolites—Biological implications of alkyl chain length. Pflug. Arch..

[143] Wang W., Xiang P., Chew W.S., Torta F., Bandla A., Lopez V., Seow W.L., Lam B.W.S., Chang J.K., Wong P. (2020). Activation of sphingosine 1-phosphate receptor 2 attenuates chemotherapy-induced neuropathy. J. Biol. Chem..

[144] Riley R.T., Merrill A.H. (2019). Ceramide synthase inhibition by fumonisins: A perfect storm of perturbed sphingolipid metabolism, signaling, and disease. J. Lipid Res..

[145] Troupiotis-Tsailaki A., Zachmann J., Gonzalez-Gil I., Gonzalez A., Ortega-Gutierrez S., Lopez-Rodriguez M.L., Pardo L., Govaerts C. (2017). Ligand chain length drives activation of lipid G protein-coupled receptors. Sci. Rep..

[146] Bu S., Kapanadze B., Hsu T., Trojanowska M. (2008). Opposite effects of dihydrosphingosine 1-phosphate and sphingosine 1-phosphate on transforming growth factor-β/Smad signaling are mediated through the PTEN/PPM1A-dependent pathway. J. Biol. Chem..

[147] Vutukuri R., Koch A., Trautmann S., Schreiber Y., Thomas D., Mayser F., Meyer Zu Heringdorf D., Pfeilschifter J., Pfeilschifter W., Brunkhorst R. (2020). S1P d20:1, an endogenous modulator of S1P d18:1/S1P(2)-dependent signaling. FASEB J..

[148] Candelore M.R., Wright M.J., Tota L.M., Milligan J., Shei G.J., Bergstrom J.D., Mandala S.M. (2002). Phytosphingosine 1-phosphate: A high affinity ligand for the S1P(4)/Edg-6 receptor. Biochem. Biophys. Res. Commun..

[149] Inagaki Y., Pham T.T., Fujiwara Y., Kohno T., Osborne D.A., Igarashi Y., Tigyi G., Parrill A.L. (2005). Sphingosine 1-phosphate analogue recognition and selectivity at S1P4 within the endothelial differentiation gene family of receptors. Biochem. J..

[150] Fahy E., Subramaniam S., Brown H.A., Glass C.K., Merrill A.H., Murphy R.C., Raetz C.R., Russell D.W., Seyama Y., Shaw W. (2005). A comprehensive classification system for lipids. J. Lipid Res..

[151] Van Overloop H., Denizot Y., Baes M., Van Veldhoven P.P. (2007). On the presence of C2-ceramide in mammalian tissues: Possible relationship to etherphospholipids and phosphorylation by ceramide kinase. Biol. Chem..

[152] Sandhoff R. (2010). Very long chain sphingolipids: Tissue expression, function and synthesis. FEBS Lett..

[153] Ohno Y., Nakamura T., Iwasaki T., Katsuyama A., Ichikawa S., Kihara A. (2023). Determining the structure of protein-bound ceramides, essential lipids for skin barrier function. iScience.

[154] Kim J.L., Mestre B., Shin S.H., Futerman A.H. (2021). Ceramide synthases: Reflections on the impact of Dr. Lina M. Obeid. Cell Signal.

[155] Goto A., Sakai S., Mizuike A., Yamaji T., Hanada K. (2022). Compartmentalization of casein kinase 1 γ CSNK1G controls the intracellular trafficking of ceramide. iScience.

[156] Hannun Y.A., Obeid L.M. (2018). Sphingolipids and their metabolism in physiology and disease. Nat. Rev. Mol. Cell Biol..

[157] Nixon G.F., Mathieson F.A., Hunter I. (2008). The multi-functional role of sphingosylphosphorylcholine. Prog. Lipid Res..

[158] van Eijk M., Ferraz M.J., Boot R.G., Aerts J. (2020). Lyso-glycosphingolipids: Presence and consequences. Essays Biochem..

[159] Dubot P., Astudillo L., Therville N., Carrie L., Pettazzoni M., Cheillan D., Stirnemann J., Levade T., Andrieu-Abadie N., Sabourdy F. (2022). Potential Role of Sphingolipidoses-Associated Lysosphingolipids in Cancer. Cancers.

[160] Stauffer B.B., Yu C. (2022). Plasma Lysosphingolipid Biomarker Measurement by Liquid Chromatography Tandem Mass Spectrometry. Methods Mol. Biol..

[161] Meyer zu Heringdorf D., Jakobs K.H. (2007). Lysophospholipid receptors: Signalling, pharmacology and regulation by lysophospholipid metabolism. Biochim. Biophys. Acta.

[162] Timm T., Hild C., Liebisch G., Rickert M., Lochnit G., Steinmeyer J. (2024). Functional Insights into the Sphingolipids C1P, S1P, and SPC in Human Fibroblast-like Synoviocytes by Proteomic Analysis. Int. J. Mol. Sci..

[163] Li G., Hu R., Kamijo Y., Nakajima T., Aoyama T., Inoue T., Node K., Kannagi R., Kyogashima M., Hara A. (2007). Establishment of a quantitative, qualitative, and high-throughput analysis of sulfatides from small amounts of sera by matrix-assisted laser desorption ionization—Time of flight mass spectrometry. Anal. Biochem..

[164] Giese A.K., Mascher H., Grittner U., Eichler S., Kramp G., Lukas J., te Vruchte D., Al Eisa N., Cortina-Borja M., Porter F.D. (2015). A novel, highly sensitive and specific biomarker for Niemann-Pick type C1 disease. Orphanet J. Rare Dis..

[165] Kubaski F., Burlina A., Pereira D., Silva C., Herbst Z.M., Trapp F.B., Michelin-Tirelli K., Lopes F.F., Burin M.G., Brusius-Facchin A.C. (2022). Quantification of lysosphingomyelin and lysosphingomyelin-509 for the screening of acid sphingomyelinase deficiency. Orphanet J. Rare Dis..

[166] Maekawa M., Jinnoh I., Matsumoto Y., Narita A., Mashima R., Takahashi H., Iwahori A., Saigusa D., Fujii K., Abe A. (2019). Structural Determination of Lysosphingomyelin-509 and Discovery of Novel Class Lipids from Patients with Niemann-Pick Disease Type C. Int. J. Mol. Sci..

[167] Sidhu R., Mondjinou Y., Qian M., Song H., Kumar A.B., Hong X., Hsu F.F., Dietzen D.J., Yanjanin N.M., Porter F.D. (2019). N-acyl-O-phosphocholineserines: Structures of a novel class of lipids that are biomarkers for Niemann-Pick C1 disease. J. Lipid Res..

[168] Sacket S.J., Im D.S. (2008). Discovery of sphingosine 1-O-methyltransferase in rat kidney and liver homogenates. Acta Pharmacol. Sin..

[169] Carter H.E., Nalbandov O., Tavormina P.A. (1951). Biochemistry of the sphingolipides. VI. The o-methyl ethers of sphingosine. J. Biol. Chem..

[170] Kisic A., Tsuda M., Kulmacz R.J., Wilson W.K., Schroepfer G.J. (1995). Sphingolipid bases. A revisitation of the O-methyl derivatives of sphingosine. Isolation and characterization of diacetate derivatives, with revised 13C nuclear magnetic resonance assignments for D-erythro-sphingosine. J. Lipid Res..

[171] Igarashi Y., Hakomori S. (1989). Enzymatic synthesis of N,N-dimethyl-sphingosine: Demonstration of the sphingosine: N-methyltransferase in mouse brain. Biochem. Biophys. Res. Commun..

[172] Igarashi Y., Kitamura K., Toyokuni T., Dean B., Fenderson B., Ogawass T., Hakomori S. (1990). A specific enhancing effect of N,N-dimethylsphingosine on epidermal growth factor receptor autophosphorylation. Demonstration of its endogenous occurrence (and the virtual absence of unsubstituted sphingosine) in human epidermoid carcinoma A431 cells. J. Biol. Chem..

[173] Morales P.R., Dillehay D.L., Moody S.J., Pallas D.C., Pruett S., Allgood J.C., Symolon H., Merrill A.H. (2007). Safingol toxicology after oral administration to TRAMP mice: Demonstration of safingol uptake and metabolism by N-acylation and N-methylation. Drug Chem. Toxicol..

[174] Symolon H., Bushnev A., Peng Q., Ramaraju H., Mays S.G., Allegood J.C., Pruett S.T., Sullards M.C., Dillehay D.L., Liotta D.C. (2011). Enigmol: A novel sphingolipid analogue with anticancer activity against cancer cell lines and in vivo models for intestinal and prostate cancer. Mol. Cancer Ther..

[175] Igarashi Y., Hakomori S., Toyokuni T., Dean B., Fujita S., Sugimoto M., Ogawa T., el-Ghendy K., Racker E. (1989). Effect of chemically well-defined sphingosine and its N-methyl derivatives on protein kinase C and src kinase activities. Biochemistry.

[176] Yatomi Y., Ruan F., Megidish T., Toyokuni T., Hakomori S., Igarashi Y. (1996). N,N-dimethylsphingosine inhibition of sphingosine kinase and sphingosine 1-phosphate activity in human platelets. Biochemistry.

[177] Prell A., Wigger D., Huwiler A., Schumacher F., Kleuser B. (2024). The sphingosine kinase 2 inhibitors ABC294640 and K145 elevate (dihydro)sphingosine 1-phosphate levels in various cells. J. Lipid Res..

[178] Patti G.J., Yanes O., Shriver L.P., Courade J.P., Tautenhahn R., Manchester M., Siuzdak G. (2012). Metabolomics implicates altered sphingolipids in chronic pain of neuropathic origin. Nat. Chem. Biol..

[179] Chen Y.J., Hill S., Huang H., Taraboletti A., Cho K., Gallo R., Manchester M., Shriver L.P., Patti G.J. (2014). Inflammation triggers production of dimethylsphingosine from oligodendrocytes. Neuroscience.

[180] Wang J., Zheng G., Wang L., Meng L., Ren J., Shang L., Li D., Bao Y. (2024). Dysregulation of sphingolipid metabolism in pain. Front. Pharmacol..

[181] Abaoui M., Boutin M., Lavoie P., Auray-Blais C. (2016). Tandem mass spectrometry multiplex analysis of methylated and non-methylated urinary Gb3 isoforms in Fabry disease patients. Clin. Chim. Acta.

[182] Merrill A.H., Sullards M.C. (2017). Opinion article on lipidomics: Inherent challenges of lipidomic analysis of sphingolipids. Biochim. Biophys. Acta Mol. Cell Biol. Lipids.

[183] Murphy R.C., Axelsen P.H. (2011). Mass spectrometric analysis of long-chain lipids. Mass Spectrom. Rev..

[184] Shaner R.L., Allegood J.C., Park H., Wang E., Kelly S., Haynes C.A., Sullards M.C., Merrill A.H. (2009). Quantitative analysis of sphingolipids for lipidomics using triple quadrupole and quadrupole linear ion trap mass spectrometers. J. Lipid Res..

[185] Panevska A., Skocaj M., Krizaj I., Macek P., Sepcic K. (2019). Ceramide phosphoethanolamine, an enigmatic cellular membrane sphingolipid. Biochim. Biophys. Acta Biomembr..

[186] Cappuccio G., Khalil S.M., Osenberg S., Li F., Maletic-Savatic M. (2023). Mass spectrometry imaging as an emerging tool for studying metabolism in human brain organoids. Front. Mol. Biosci..

[187] Vacaru A.M., Tafesse F.G., Ternes P., Kondylis V., Hermansson M., Brouwers J.F., Somerharju P., Rabouille C., Holthuis J.C. (2009). Sphingomyelin synthase-related protein SMSr controls ceramide homeostasis in the ER. J. Cell Biol..

[188] Bickert A., Ginkel C., Kol M., vom Dorp K., Jastrow H., Degen J., Jacobs R.L., Vance D.E., Winterhager E., Jiang X.C. (2015). Functional characterization of enzymes catalyzing ceramide phosphoethanolamine biosynthesis in mice. J. Lipid Res..

[189] Cabukusta B., Nettebrock N.T., Kol M., Hilderink A., Tafesse F.G., Holthuis J.C.M. (2017). Ceramide phosphoethanolamine synthase SMSr is a target of caspase-6 during apoptotic cell death. Biosci. Rep..

[190] Hu K., Zhang Q., Chen Y., Yang J., Xia Y., Rao B., Li S., Shen Y., Cao M., Lu H. (2024). Cryo-EM structure of human sphingomyelin synthase and its mechanistic implications for sphingomyelin synthesis. Nat. Struct. Mol. Biol..

[191] Sakane F., Murakami C., Sakai H. (2024). Upstream and downstream pathways of diacylglycerol kinase: Novel phosphatidylinositol turnover-independent signal transduction pathways. Adv. Biol. Regul..

[192] Presa N., Gomez-Larrauri A., Dominguez-Herrera A., Trueba M., Gomez-Munoz A. (2020). Novel signaling aspects of ceramide 1-phosphate. Biochim. Biophys. Acta Mol. Cell Biol. Lipids.

[193] Yamazaki A., Kawashima A., Honda T., Kohama T., Murakami C., Sakane F., Murayama T., Nakamura H. (2023). Identification and characterization of diacylglycerol kinase ζ as a novel enzyme producing ceramide-1-phosphate. Biochim. Biophys. Acta Mol. Cell Biol. Lipids.

[194] Lachmayr H., Merrill A.H. (2024). A Brief Overview of the Toxic Sphingomyelinase Ds of Brown Recluse Spider Venom and Other Organisms and Simple Methods To Detect Production of Its Signature Cyclic Ceramide Phosphate. Mol. Pharmacol..

[195] Boudker O., Futerman A.H. (1993). Detection and characterization of ceramide-1-phosphate phosphatase activity in rat liver plasma membrane. J. Biol. Chem..

[196] Park Y., Jang J., Lee J., Baek H., Park J., Cha S.R., Lee S.B., Na S., Kwon J.W., Hong S.H. (2023). Cyclic Phytosphingosine-1-Phosphate Primed Mesenchymal Stem Cells Ameliorate LPS-Induced Acute Lung Injury in Mice. Int. J. Stem Cells.

[197] Lee H.J., Choe K., Park J.S., Khan A., Kim M.W., Park T.J., Kim M.O. (2022). O-Cyclic Phytosphingosine-1-Phosphate Protects against Motor Dysfunctions and Glial Cell Mediated Neuroinflammation in the Parkinson’s Disease Mouse Models. Antioxidants.

[198] Heiles S., Kompauer M., Muller M.A., Spengler B. (2020). Atmospheric-Pressure MALDI Mass Spectrometry Imaging at 213 nm Laser Wavelength. J. Am. Soc. Mass Spectrom..

[199] Bhaduri A., Neumann E.K., Kriegstein A.R., Sweedler J.V. (2021). Identification of Lipid Heterogeneity and Diversity in the Developing Human Brain. JACS Au.

[200] Guimaraes L.L., Toledo M.S., Ferreira F.A., Straus A.H., Takahashi H.K. (2014). Structural diversity and biological significance of glycosphingolipids in pathogenic and opportunistic fungi. Front. Cell Infect. Microbiol..

[201] Serrano A.A., Schenkman S., Yoshida N., Mehlert A., Richardson J.M., Ferguson M.A. (1995). The lipid structure of the glycosylphosphatidylinositol-anchored mucin-like sialic acid acceptors of Trypanosoma cruzi changes during parasite differentiation from epimastigotes to infective metacyclic trypomastigote forms. J. Biol. Chem..

[202] Levatti E.V.D.C., Toledo M.S., Costa R.W., Bahia D., Mortara R.A., Takahashi H.K., Straus A.H. (2017). Leishmania (Viannia) braziliensis Inositol Phosphorylceramide: Distinctive Sphingoid Base Composition. Front. Microbiol..

[203] Cummings R.D. (2023). Glycosphingolipids in human parasites. FEBS Open Bio.

[204] Giorgi M.E., Lederkremer R.M. (2020). The Glycan Structure of T. cruzi mucins Depends on the Host. Insights on the Chameleonic Galactose. Molecules.

[205] Matos G.S., Fernandes C.M., Del Poeta M. (2023). Role of sphingolipids in the host-pathogen interaction. Biochim. Biophys. Acta Mol. Cell Biol. Lipids.

[206] Alpizar-Sosa E.A., Zimbres F.M., Mantilla B.S., Dickie E.A., Wei W., Burle-Caldas G.A., Filipe L.N.S., Van Bocxlaer K., Price H.P., Ibarra-Meneses A.V. (2024). Evaluation of the Leishmania Inositol Phosphorylceramide Synthase as a Drug Target Using a Chemical and Genetic Approach. ACS Infect. Dis..

[207] Schnaar R.L., Sandhoff R., Tiemeyer M., Kinoshita T., Varki A., Cummings R.D., Esko J.D., Stanley P., Hart G.W., Aebi M., Mohnen D., Kinoshita T., Packer N.H., Prestegard J.H. (2022). Glycosphingolipids. Essentials of Glycobiology.

[208] Chiricozzi E., Aureli M., Mauri L., Di Biase E., Lunghi G., Fazzari M., Valsecchi M., Carsana E.V., Loberto N., Prinetti A. (2021). Glycosphingolipids. Adv. Exp. Med. Biol..

[209] Guo Z. (2022). The Structural Diversity of Natural Glycosphingolipids (GSLs). J. Carbohydr. Chem..

[210] Lunghi G., Fazzari M., Di Biase E., Mauri L., Chiricozzi E., Sonnino S. (2021). The structure of gangliosides hides a code for determining neuronal functions. FEBS Open Bio.

[211] Schengrund C.L. (2024). Sphingolipids: Less Enigmatic but Still Many Questions about the Role(s) of Ceramide in the Synthesis/Function of the Ganglioside Class of Glycosphingolipids. Int. J. Mol. Sci..

[212] Farwanah H., Kolter T. (2012). Lipidomics of glycosphingolipids. Metabolites.

[213] Horejsi K., Jirasko R., Chocholouskova M., Wolrab D., Kahoun D., Holcapek M. (2021). Comprehensive Identification of Glycosphingolipids in Human Plasma Using Hydrophilic Interaction Liquid Chromatography—Electrospray Ionization Mass Spectrometry. Metabolites.

[214] Horejsi K., Holcapek M. (2024). Unraveling the complexity of glycosphingolipidome: The key role of mass spectrometry in the structural analysis of glycosphingolipids. Anal. Bioanal. Chem..

[215] Vance D.E., Krivit W., Sweeley C.C. (1969). Concentrations of glycosyl ceramides in plasma and red cells in Fabry’s disease, a glycolipid lipidosis. J. Lipid Res..

[216] Touboul D., Roy S., Germain D.P., Baillet A., Brion F., Prognon P., Chaminade P., Laprevote O. (2005). Fast fingerprinting by MALDI-TOF mass spectrometry of urinary sediment glycosphingolipids in Fabry disease. Anal. Bioanal. Chem..

[217] von Gerichten J., Schlosser K., Lamprecht D., Morace I., Eckhardt M., Wachten D., Jennemann R., Grone H.J., Mack M., Sandhoff R. (2017). Diastereomer-specific quantification of bioactive hexosylceramides from bacteria and mammals. J. Lipid Res..

[218] von Gerichten J., Lamprecht D., Opalka L., Soulard D., Marsching C., Pilz R., Sencio V., Herzer S., Galy B., Nordstrom V. (2019). Bacterial immunogenic α-galactosylceramide identified in the murine large intestine: Dependency on diet and inflammation. J. Lipid Res..

[219] Okino N., Li M., Qu Q., Nakagawa T., Hayashi Y., Matsumoto M., Ishibashi Y., Ito M. (2020). Two bacterial glycosphingolipid synthases responsible for the synthesis of glucuronosylceramide and α-galactosylceramide. J. Biol. Chem..

[220] Ustjanzew A., Sencio V., Trottein F., Faber J., Sandhoff R., Paret C. (2022). Interaction between Bacteria and the Immune System for Cancer Immunotherapy: The alpha-GalCer Alliance. Int. J. Mol. Sci..

[221] Xu H., Boucher F.R., Nguyen T.T., Taylor G.P., Tomlinson J.J., Ortega R.A., Simons B., Schlossmacher M.G., Saunders-Pullman R., Shaw W. (2019). DMS as an orthogonal separation to LC/ESI/MS/MS for quantifying isomeric cerebrosides in plasma and cerebrospinal fluid. J. Lipid Res..

[222] Takahashi H., Perez-Canamas A., Lee C.W., Ye H., Han X., Strittmatter S.M. (2024). Lysosomal TMEM106B interacts with galactosylceramidase to regulate myelin lipid metabolism. Commun. Biol..

[223] Mauri L., Sonnino S. (2023). Alkali-labile gangliosides. Glycoconj. J..

[224] Klenk E., Lohr J.P. (1967). [On the ester cerebrosides of brain]. Hoppe Seylers Z. Physiol. Chem..

[225] Dasgupta S., Levery S.B., Hogan E.L. (2002). 3-O-acetyl-sphingosine-series myelin glycolipids: Characterization of novel 3-O-acetyl-sphingosine galactosylceramide. J. Lipid Res..

[226] Bennion B., Dasgupta S., Hogan E.L., Levery S.B. (2007). Characterization of novel myelin components 3-O-acetyl-sphingosine galactosylceramides by electrospray ionization Q-TOF MS and MS/CID-MS of Li+ adducts. J. Mass. Spectrom..

[227] Theret N., Boulenguer P., Wieruszeski J.M., Fournet B., Fruchart J.C., Bourre J.M., Delbart C. (1987). Structure determination of the polymorphism of acylgalactosylceramide in rat brain by gas chromatography/mass spectrometry and proton magnetic resonance. Biochim. Biophys. Acta.

[228] Podbielska M., Dasgupta S., Levery S.B., Tourtellotte W.W., Annuk H., Moran A.P., Hogan E.L. (2010). Novel myelin penta- and hexa-acetyl-galactosyl-ceramides: Structural characterization and immunoreactivity in cerebrospinal fluid. J. Lipid Res..

[229] Podbielska M., Levery S.B., Hogan E.L. (2011). The structural and functional role of myelin fast-migrating cerebrosides: Pathological importance in multiple sclerosis. Clin. Lipidol..

[230] Gately C.M., Podbielska M., Counihan T., Hennessy M., Leahy T., Moran A.P., Hogan E.L., O’Keeffe J. (2013). Invariant Natural Killer T-cell anergy to endogenous myelin acetyl-glycolipids in multiple sclerosis. J. Neuroimmunol..

[231] Levery S.B., Nudelman E.D., Hakomori S. (1992). Novel modification of glycosphingolipids by long-chain cyclic acetals: Isolation and characterization of plasmalocerebroside from human brain. Biochemistry.

[232] Nudelman E.D., Levery S.B., Igarashi Y., Hakomori S. (1992). Plasmalopsychosine, a novel plasmal (fatty aldehyde) conjugate of psychosine with cyclic acetal linkage. Isolation and characterization from human brain white matter. J. Biol. Chem..

[233] Cavdarli S., Delannoy P., Groux-Degroote S. (2020). O-acetylated Gangliosides as Targets for Cancer Immunotherapy. Cells.

[234] Sarkar A., Banerjee S., Biswas K. (2023). Multi-dimensional role of gangliosides in modulating cancer hallmarks and their prospects in targeted cancer therapy. Front. Pharmacol..

[235] Aureli M., Samarani M., Loberto N., Bassi R., Murdica V., Prioni S., Prinetti A., Sonnino S. (2014). The glycosphingolipid hydrolases in the central nervous system. Mol. Neurobiol..

[236] Wang J., Shewell L.K., Day C.J., Jennings M.P. (2023). N-glycolylneuraminic acid as a carbohydrate cancer biomarker. Transl. Oncol..

[237] Jahan M., Thomson P.C., Wynn P.C., Wang B. (2023). Red Meat Derived Glycan, N-acetylneuraminic Acid (Neu5Ac) Is a Major Sialic Acid in Different Skeletal Muscles and Organs of Nine Animal Species-A Guideline for Human Consumers. Foods.

[238] Samraj A.N., Pearce O.M., Laubli H., Crittenden A.N., Bergfeld A.K., Banda K., Gregg C.J., Bingman A.E., Secrest P., Diaz S.L. (2015). A red meat-derived glycan promotes inflammation and cancer progression. Proc. Natl. Acad. Sci. USA.

[239] Riboni L., Sonnino S., Acquotti D., Malesci A., Ghidoni R., Egge H., Mingrino S., Tettamanti G. (1986). Natural occurrence of ganglioside lactones. Isolation and characterization of GD1b inner ester from adult human brain. J. Biol. Chem..

[240] Bosslet K., Mennel H.D., Rodden F., Bauer B.L., Wagner F., Altmannsberger A., Sedlacek H.H., Wiegandt H. (1989). Monoclonal antibodies against epitopes on ganglioside GD2 and its lactones. Markers for gliomas and neuroblastomas. Cancer Immunol. Immunother..

[241] Rabionet M., Bayerle A., Marsching C., Jennemann R., Grone H.J., Yildiz Y., Wachten D., Shaw W., Shayman J.A., Sandhoff R. (2013). 1-O-acylceramides are natural components of human and mouse epidermis. J. Lipid Res..

[242] Senkal C.E., Salama M.F., Snider A.J., Allopenna J.J., Rana N.A., Koller A., Hannun Y.A., Obeid L.M. (2017). Ceramide Is Metabolized to Acylceramide and Stored in Lipid Droplets. Cell Metab..

[243] Lin M.H., Miner J.H., Turk J., Hsu F.F. (2017). Linear ion-trap MS(n) with high-resolution MS reveals structural diversity of 1-O-acylceramide family in mouse epidermis. J. Lipid Res..

[244] Shayman J.A., Abe A., Hiraoka M. (2004). A turn in the road: How studies on the pharmacology of glucosylceramide synthase inhibitors led to the identification of a lysosomal phospholipase A2 with ceramide transacylase activity. Glycoconj. J..

[245] Hernandez-Corbacho M., Canals D. (2024). Drug Targeting of Acyltransferases in the Triacylglyceride and 1-O-AcylCeramide Biosynthetic Pathways. Mol. Pharmacol..

[246] Hinkovska-Galcheva V., Treadwell T., Shillingford J.M., Lee A., Abe A., Tesmer J.J.G., Shayman J.A. (2021). Inhibition of lysosomal phospholipase A2 predicts drug-induced phospholipidosis. J. Lipid Res..

[247] Hernandez-Corbacho M.J., Obeid L.M. (2019). A novel role for DGATs in cancer. Adv. Biol. Regul..

[248] Mathiowetz A.J., Olzmann J.A. (2024). Lipid droplets and cellular lipid flux. Nat. Cell Biol..

[249] Wertz P.W. (2021). Lipid Metabolic Events Underlying the Formation of the Corneocyte Lipid Envelope. Ski. Pharmacol. Physiol..

[250] Yamamoto Y., Sassa T., Kihara A. (2024). Comparison of skin barrier abnormalities and epidermal ceramide profiles among three ω-O-acylceramide synthesis-deficient mouse strains. J. Dermatol. Sci..

[251] Wertz P.W. (2023). Linoleate-Containing Acylglucosylceramide, Acylceramide, and Events Associated with Formation of the Epidermal Permeability Barrier. Ski. Pharmacol. Physiol..

[252] Sakamaki J.I., Mizushima N. (2023). Cell biology of protein-lipid conjugation. Cell Struct. Funct..

[253] Russell L.J., DiGiovanna J.J., Rogers G.R., Steinert P.M., Hashem N., Compton J.G., Bale S.J. (1995). Mutations in the gene for transglutaminase 1 in autosomal recessive lamellar ichthyosis. Nat. Genet..

[254] Conzelmann A., Puoti A., Lester R.L., Desponds C. (1992). Two different types of lipid moieties are present in glycophosphoinositol-anchored membrane proteins of Saccharomyces cerevisiae. EMBO J..

[255] Nakayasu E.S., Yashunsky D.V., Nohara L.L., Torrecilhas A.C., Nikolaev A.V., Almeida I.C. (2009). GPIomics: Global analysis of glycosylphosphatidylinositol-anchored molecules of Trypanosoma cruzi. Mol. Syst. Biol..

[256] Borges A.R., Link F., Engstler M., Jones N.G. (2021). The Glycosylphosphatidylinositol Anchor: A Linchpin for Cell Surface Versatility of Trypanosomatids. Front. Cell Dev. Biol..

[257] Dos Santos N.S.A., Estevez-Castro C.F., Macedo J.P., Chame D.F., Castro-Gomes T., Santos-Cardoso M., Burle-Caldas G.A., Covington C.N., Steel P.G., Smith T.K. (2023). Disruption of the inositol phosphorylceramide synthase gene affects Trypanosoma cruzi differentiation and infection capacity. PLoS Negl. Trop. Dis..

[258] Ostroumova O.S., Efimova S.S. (2023). Lipid-Centric Approaches in Combating Infectious Diseases: Antibacterials, Antifungals and Antivirals with Lipid-Associated Mechanisms of Action. Antibiotics.

[259] Martinez-Gardeazabal J., Roman E.G.d.S., Moreno-Rodriguez M., Llorente-Ovejero A., Manuel I., Rodriguez-Puertas R. (2017). Lipid mapping of the rat brain for models of disease. Biochim. Biophys. Acta Biomembr..

[260] Leontyev D., Pulliam A.N., Ma X., Gaul D.A., LaPlaca M.C., Fernandez F.M. (2024). Spatial lipidomics maps brain alterations associated with mild traumatic brain injury. Front. Chem..

[261] Snook C.F., Jones J.A., Hannun Y.A. (2006). Sphingolipid-binding proteins. Biochim. Biophys. Acta.

[262] Haberkant P., Stein F., Hoglinger D., Gerl M.J., Brugger B., Van Veldhoven P.P., Krijgsveld J., Gavin A.C., Schultz C. (2016). Bifunctional Sphingosine for Cell-Based Analysis of Protein-Sphingolipid Interactions. ACS Chem. Biol..

[263] Russo D., Parashuraman S., D’Angelo G. (2016). Glycosphingolipid-Protein Interaction in Signal Transduction. Int. J. Mol. Sci..

[264] Ledeen R.W., Kopitz J., Abad-Rodriguez J., Gabius H.J. (2018). Glycan Chains of Gangliosides: Functional Ligands for Tissue Lectins (Siglecs/Galectins). Prog. Mol. Biol. Transl. Sci..

[265] Azzaz F., Yahi N., Di Scala C., Chahinian H., Fantini J. (2022). Ganglioside binding domains in proteins: Physiological and pathological mechanisms. Adv. Protein Chem. Struct. Biol..

[266] Capelluto D.G.S. (2022). The repertoire of protein-sulfatide interactions reveal distinct modes of sulfatide recognition. Front. Mol. Biosci..

[267] Smith E.R., Merrill A.H. (1995). Differential roles of de novo sphingolipid biosynthesis and turnover in the “burst” of free sphingosine and sphinganine, and their 1-phosphates and N-acyl-derivatives, that occurs upon changing the medium of cells in culture. J. Biol. Chem..

[268] Schroeder J.J., Crane H.M., Xia J., Liotta D.C., Merrill A.H. (1994). Disruption of sphingolipid metabolism and stimulation of DNA synthesis by fumonisin B1. A molecular mechanism for carcinogenesis associated with Fusarium moniliforme. J. Biol. Chem..

[269] Lavie Y., Blusztajn J.K., Liscovitch M. (1994). Formation of endogenous free sphingoid bases in cells induced by changing medium conditions. Biochim. Biophys. Acta.

[270] Merrill A.H., Wang E., Mullins R.E. (1988). Kinetics of long-chain (sphingoid) base biosynthesis in intact LM cells: Effects of varying the extracellular concentrations of serine and fatty acid precursors of this pathway. Biochemistry.

[271] Messmer T.O., Wang E., Stevens V.L., Merrill A.H. (1989). Sphingolipid biosynthesis by rat liver cells: Effects of serine, fatty acids and lipoproteins. J. Nutr..

[272] Harrison P.J., Gable K., Somashekarappa N., Kelly V., Clarke D.J., Naismith J.H., Dunn T.M., Campopiano D.J. (2019). Use of isotopically labeled substrates reveals kinetic differences between human and bacterial serine palmitoyltransferase. J. Lipid Res..

[273] Levin-Konigsberg R., Mitra K., Spees K., Nigam A., Liu K., Januel C., Hivare P., Arana S.M., Prolo L.M., Kundaje A. (2024). An SLC12A9-dependent ion transport mechanism maintains lysosomal osmolarity. Dev. Cell.

[274] Breiden B., Sandhoff K. (2019). Emerging mechanisms of drug-induced phospholipidosis. Biol. Chem..

[275] Warden L.A., Menaldino D.S., Wilson T., Liotta D.C., Smith E.R., Merrill A.H. (1999). Identification of ammonium ion and 2,6-bis(ω-aminobutyl)-3, 5-diiminopiperazine as endogenous factors that account for the “burst” of sphingosine upon changing the medium of J774 cells in culture. J. Biol. Chem..

[276] Holecek M. (2022). Serine Metabolism in Health and Disease and as a Conditionally Essential Amino Acid. Nutrients.

[277] Handzlik M.K., Metallo C.M. (2023). Sources and Sinks of Serine in Nutrition, Health, and Disease. Annu. Rev. Nutr..

[278] Smith E.R., Jones P.L., Boss J.M., Merrill A.H. (1997). Changing J774A.1 cells to new medium perturbs multiple signaling pathways, including the modulation of protein kinase C by endogenous sphingoid bases. J. Biol. Chem..

[279] Kandalgaonkar M.R., Kumar V., Vijay-Kumar M. (2024). Digestive dynamics: Unveiling interplay between the gut microbiota and the liver in macronutrient metabolism and hepatic metabolic health. Physiol. Rep..

[280] Nishiyama A., Yokote Y., Sakagami H. (2010). Changes in amino acid metabolism during activation of mouse macrophage-like cell lines. In Vivo.

[281] Sakagami H., Kishino K., Amano O., Kanda Y., Kunii S., Yokote Y., Oizumi H., Oizumi T. (2009). Cell death induced by nutritional starvation in mouse macrophage-like RAW264.7 cells. Anticancer Res..

[282] Wang E., Norred W.P., Bacon C.W., Riley R.T., Merrill A.H. (1991). Inhibition of sphingolipid biosynthesis by fumonisins. Implications for diseases associated with Fusarium moniliforme. J. Biol. Chem..

[283] Zhang Z., Fang Q., Xie T., Gong X. (2024). Mechanism of ceramide synthase inhibition by fumonisin B(1). Structure.

[284] Pascoa T.C., Pike A.C.W., Tautermann C.S., Chi G., Traub M., Quigley A., Chalk R., Stefanic S., Thamm S., Pautsch A. (2024). Structural basis of the mechanism and inhibition of a human ceramide synthase. Nat. Struct. Mol. Biol..

[285] Schafer J.H., Clausmeyer L., Korner C., Esch B.M., Wolf V.N., Sapia J., Ahmed Y., Walter S., Vanni S., Januliene D. (2024). Structure of the yeast ceramide synthase. Nat. Struct. Mol. Biol..

[286] Merrill A.H., van Echten G., Wang E., Sandhoff K. (1993). Fumonisin B1 inhibits sphingosine (sphinganine) N-acyltransferase and de novo sphingolipid biosynthesis in cultured neurons in situ. J. Biol. Chem..

[287] Riley R.T., An N.H., Showker J.L., Yoo H.S., Norred W.P., Chamberlain W.J., Wang E., Merrill A.H., Motelin G., Beasley V.R. (1993). Alteration of tissue and serum sphinganine to sphingosine ratio: An early biomarker of exposure to fumonisin-containing feeds in pigs. Toxicol. Appl. Pharmacol..

[288] Riley R.T., Torres O., Matute J., Gregory S.G., Ashley-Koch A.E., Showker J.L., Mitchell T., Voss K.A., Maddox J.R., Gelineau-van Waes J.B. (2015). Evidence for fumonisin inhibition of ceramide synthase in humans consuming maize-based foods and living in high exposure communities in Guatemala. Mol. Nutr. Food Res..

[289] Green C.D., Maceyka M., Cowart L.A., Spiegel S. (2021). Sphingolipids in metabolic disease: The good, the bad, and the unknown. Cell Metab..

[290] Kano K., Aoki J., Hla T. (2022). Lysophospholipid Mediators in Health and Disease. Annu. Rev. Pathol..

[291] Humpf H.U., Schmelz E.M., Meredith F.I., Vesper H., Vales T.R., Wang E., Menaldino D.S., Liotta D.C., Merrill A.H. (1998). Acylation of naturally occurring and synthetic 1-deoxysphinganines by ceramide synthase. Formation of N-palmitoyl-aminopentol produces a toxic metabolite of hydrolyzed fumonisin, AP1, and a new category of ceramide synthase inhibitor. J. Biol. Chem..

[292] Merrill A.H., Schmelz E.M., Wang E., Dillehay D.L., Rice L.G., Meredith F., Riley R.T. (1997). Importance of sphingolipids and inhibitors of sphingolipid metabolism as components of animal diets. J. Nutr..

[293] Zhou G., Hu S., Xie L., Huang H., Huang W., Zheng Q., Zhang N. (2024). Individual and combined occurrences of the prevalent mycotoxins in commercial feline and canine food. Mycotoxin Res..

[294] Afrin F., Mateen S., Oman J., Lai J.C.K., Barrott J.J., Pashikanti S. (2023). Natural Products and Small Molecules Targeting Cellular Ceramide Metabolism to Enhance Apoptosis in Cancer Cells. Cancers.

[295] Li Y., Talbot C.L., Chandravanshi B., Ksiazek A., Sood A., Chowdhury K.H., Maschek J.A., Cox J., Babu A.K.S., Paz H.A. (2022). Cordyceps inhibits ceramide biosynthesis and improves insulin resistance and hepatic steatosis. Sci. Rep..

[296] Michel C., van Echten-Deckert G., Rother J., Sandhoff K., Wang E., Merrill A.H. (1997). Characterization of ceramide synthesis. A dihydroceramide desaturase introduces the 4,5-trans-double bond of sphingosine at the level of dihydroceramide. J. Biol. Chem..

[297] Fabrias G., Munoz-Olaya J., Cingolani F., Signorelli P., Casas J., Gagliostro V., Ghidoni R. (2012). Dihydroceramide desaturase and dihydrosphingolipids: Debutant players in the sphingolipid arena. Prog. Lipid Res..

[298] Alsanafi M., Brown R.D.R., Oh J., Adams D.R., Torta F., Pyne N.J., Pyne S. (2021). Dihydroceramide Desaturase Functions as an Inducer and Rectifier of Apoptosis: Effect of Retinol Derivatives, Antioxidants and Phenolic Compounds. Cell Biochem. Biophys..

[299] Pitman M.R., Lewis A.C., Davies L.T., Moretti P.A.B., Anderson D., Creek D.J., Powell J.A., Pitson S.M. (2022). The sphingosine 1-phosphate receptor 2/4 antagonist JTE-013 elicits off-target effects on sphingolipid metabolism. Sci. Rep..

[300] Rahmaniyan M., Curley R.W., Obeid L.M., Hannun Y.A., Kraveka J.M. (2011). Identification of dihydroceramide desaturase as a direct in vitro target for fenretinide. J. Biol. Chem..

[301] Obeid L.M., Linardic C.M., Karolak L.A., Hannun Y.A. (1993). Programmed cell death induced by ceramide. Science.

[302] Blitzer J.T., Wang L., Summers S.A. (2020). DES1: A Key Driver of Lipotoxicity in Metabolic Disease. DNA Cell Biol..

[303] Jimenez de Oya N., San-Felix A., Casasampere M., Blazquez A.B., Mingo-Casas P., Escribano-Romero E., Calvo-Pinilla E., Poderoso T., Casas J., Saiz J.C. (2023). Pharmacological Elevation of Cellular Dihydrosphingomyelin Provides a Novel Antiviral Strategy against West Nile Virus Infection. Antimicrob. Agents Chemother..

[304] Spassieva S.D., Rahmaniyan M., Bielawski J., Clarke C.J., Kraveka J.M., Obeid L.M. (2012). Cell density-dependent reduction of dihydroceramide desaturase activity in neuroblastoma cells. J. Lipid Res..

[305] Liu Y., Chen Y., Momin A., Shaner R., Wang E., Bowen N.J., Matyunina L.V., Walker L.D., McDonald J.F., Sullards M.C. (2010). Elevation of sulfatides in ovarian cancer: An integrated transcriptomic and lipidomic analysis including tissue-imaging mass spectrometry. Mol. Cancer.

[306] Lopez-Montero I., Rodriguez N., Cribier S., Pohl A., Velez M., Devaux P.F. (2005). Rapid transbilayer movement of ceramides in phospholipid vesicles and in human erythrocytes. J. Biol. Chem..

[307] Contreras F.X., Sanchez-Magraner L., Alonso A., Goni F.M. (2010). Transbilayer (flip-flop) lipid motion and lipid scrambling in membranes. FEBS Lett..

[308] Liu Y. (2010). Regulation of Ceramide and Its Metabolites: Biosynthesis & In Situ Sphingolipid Analysis.

[309] Aridor M., Balch W.E. (1996). Principles of selective transport: Coat complexes hold the key. Trends Cell Biol..

[310] Fujiwara T., Oda K., Yokota S., Takatsuki A., Ikehara Y. (1988). Brefeldin A causes disassembly of the Golgi complex and accumulation of secretory proteins in the endoplasmic reticulum. J. Biol. Chem..

[311] Jarocki M., Turek K., Saczko J., Tarek M., Kulbacka J. (2024). Lipids associated with autophagy: Mechanisms and therapeutic targets. Cell Death Discov..

[312] Ohba Y., Motohashi M., Arita M. (2024). Characterization of UGT8 as a monogalactosyl diacylglycerol synthase in mammals. J. Biochem..

[313] Vesper H., Schmelz E.M., Nikolova-Karakashian M.N., Dillehay D.L., Lynch D.V., Merrill A.H. (1999). Sphingolipids in food and the emerging importance of sphingolipids to nutrition. J. Nutr..

[314] Yunoki K., Ogawa T., Ono J., Miyashita R., Aida K., Oda Y., Ohnishi M. (2008). Analysis of sphingolipid classes and their contents in meals. Biosci. Biotechnol. Biochem..

[315] Xiao X., Le H.H., Lee M.T., Lamm D., Johnson E.L., Brito I.L. (2024). Prevotella copri variants among a single host diverge in sphingolipid production. mBio.

[316] Brown E.M., Clardy J., Xavier R.J. (2023). Gut microbiome lipid metabolism and its impact on host physiology. Cell Host Microbe.

[317] Yamashita S., Kinoshita M., Miyazawa T. (2021). Dietary Sphingolipids Contribute to Health via Intestinal Maintenance. Int. J. Mol. Sci..

[318] Nilsson A., Duan R.D. (2019). Pancreatic and mucosal enzymes in choline phospholipid digestion. Am. J. Physiol. Gastrointest. Liver Physiol..

[319] Buller H.A., Van Wassenaer A.G., Raghavan S., Montgomery R.K., Sybicki M.A., Grand R.J. (1989). New insights into lactase and glycosylceramidase activities of rat lactase-phlorizin hydrolase. Am. J. Physiol..

[320] Lee M.T., Le H.H., Johnson E.L. (2021). Dietary sphinganine is selectively assimilated by members of the mammalian gut microbiome. J. Lipid Res..

[321] Nilsson A., Duan R.D. (2006). Absorption and lipoprotein transport of sphingomyelin. J. Lipid Res..

[322] Sugawara T., Kinoshita M., Ohnishi M., Tsuzuki T., Miyazawa T., Nagata J., Hirata T., Saito M. (2004). Efflux of sphingoid bases by P-glycoprotein in human intestinal Caco-2 cells. Biosci. Biotechnol. Biochem..

[323] Fujii A., Manabe Y., Aida K., Tsuduki T., Hirata T., Sugawara T. (2017). Selective Absorption of Dietary Sphingoid Bases from the Intestine via Efflux by P-Glycoprotein in Rats. J. Nutr. Sci. Vitaminol..

[324] Calzada C., Cheillan D., Ritsch N., Vors C., Durand A., Pesenti S., Pettazzoni M., Meugnier E., Michalski M.C., Penhoat A. (2024). Intestinal absorption of sphingosine: New insights on generated ceramide species using stable isotope tracing in vitro. J. Lipid Res..

[325] Narita T., Naganuma T., Sase Y., Kihara A. (2016). Long-chain bases of sphingolipids are transported into cells via the acyl-CoA synthetases. Sci. Rep..

[326] Rohrhofer J., Zwirzitz B., Selberherr E., Untersmayr E. (2021). The Impact of Dietary Sphingolipids on Intestinal Microbiota and Gastrointestinal Immune Homeostasis. Front. Immunol..

[327] Lai M.K., Chew W.S., Torta F., Rao A., Harris G.L., Chun J., Herr D.R. (2016). Biological Effects of Naturally Occurring Sphingolipids, Uncommon Variants, and Their Analogs. Neuromolecular Med..

[328] Wang X., Wang Y., Xu J., Xue C. (2021). Sphingolipids in food and their critical roles in human health. Crit. Rev. Food Sci. Nutr..

[329] Morifuji M., Higashi S., Oba C., Ichikawa S., Kawahata K., Yamaji T., Itoh H., Manabe Y., Sugawara T. (2015). Milk Phospholipids Enhance Lymphatic Absorption of Dietary Sphingomyelin in Lymph-Cannulated Rats. Lipids.

[330] Ohta K., Hiraki S., Miyanabe M., Ueki T., Aida K., Manabe Y., Sugawara T. (2021). Appearance of Intact Molecules of Dietary Ceramides Prepared from Soy Sauce Lees and Rice Glucosylceramides in Mouse Plasma. J. Agric. Food Chem..

[331] Johnson E.L., Heaver S.L., Waters J.L., Kim B.I., Bretin A., Goodman A.L., Gewirtz A.T., Worgall T.S., Ley R.E. (2020). Sphingolipids produced by gut bacteria enter host metabolic pathways impacting ceramide levels. Nat. Commun..

[332] Duan J., Sugawara T., Hirose M., Aida K., Sakai S., Fujii A., Hirata T. (2012). Dietary sphingolipids improve skin barrier functions via the upregulation of ceramide synthases in the epidermis. Exp. Dermatol..

[333] Tomonaga N., Manabe Y., Aida K., Sugawara T. (2020). Dietary ceramide 2-aminoethylphosphonate, a marine sphingophosphonolipid, improves skin barrier function in hairless mice. Sci. Rep..

[334] Park E.J., Suh M., Ramanujam K., Steiner K., Begg D., Clandinin M.T. (2005). Diet-induced changes in membrane gangliosides in rat intestinal mucosa, plasma and brain. J. Pediatr. Gastroenterol. Nutr..

[335] Zheng L., Fleith M., Giuffrida F., O’Neill B.V., Schneider N. (2019). Dietary Polar Lipids and Cognitive Development: A Narrative Review. Adv. Nutr..

[336] Wang H., Sency V., McJarrow P., Bright A., Huang Q., Cechner K., Szekely J., Brace J., Wang A., Liu D. (2019). Oral Ganglioside Supplement Improves Growth and Development in Patients with Ganglioside GM3 Synthase Deficiency. JIMD Rep..

[337] Inokuchi J.I., Go S., Suzuki A., Nakagawasai O., Odaira-Satoh T., Veillon L., Nitta T., McJarrow P., Kanoh H., Inamori K.I. (2024). Dietary gangliosides rescue GM3 synthase deficiency outcomes in mice accompanied by neurogenesis in the hippocampus. Front. Neurosci..

[338] Phung N.V., Rong F., Xia W.Y., Fan Y., Li X.Y., Wang S.A., Li F.L. (2024). Nervonic acid and its sphingolipids: Biological functions and potential food applications. Crit. Rev. Food Sci. Nutr..

[339] Norris G.H., Jiang C., Ryan J., Porter C.M., Blesso C.N. (2016). Milk sphingomyelin improves lipid metabolism and alters gut microbiota in high fat diet-fed mice. J. Nutr. Biochem..

[340] Custers, Emma E.M., Kiliaan, Amanda J. (2022). Dietary lipids from body to brain. Prog. Lipid Res..

[341] Kobayashi E., Motoki K., Uchida T., Fukushima H., Koezuka Y. (1995). KRN7000, a novel immunomodulator, and its antitumor activities. Oncol. Res..

[342] Oh S.F., Praveena T., Song H., Yoo J.S., Jung D.J., Erturk-Hasdemir D., Hwang Y.S., Lee C.C., Le Nours J., Kim H. (2022). Publisher Correction: Host immunomodulatory lipids created by symbionts from dietary amino acids. Nature.

[343] Kyriazi A.A., Karaglani M., Agelaki S., Baritaki S. (2024). Intratumoral Microbiome: Foe or Friend in Reshaping the Tumor Microenvironment Landscape?. Cells.

[344] Symolon H., Schmelz E.M., Dillehay D.L., Merrill A.H. (2004). Dietary soy sphingolipids suppress tumorigenesis and gene expression in 1,2-dimethylhydrazine-treated CF1 mice and ApcMin/+ mice. J. Nutr..

[345] Camp E.R., Patterson L.D., Kester M., Voelkel-Johnson C. (2017). Therapeutic implications of bioactive sphingolipids: A focus on colorectal cancer. Cancer Biol. Ther..

[346] Fujiwara K., Kitatani K., Fukushima K., Yazama H., Umehara H., Kikuchi M., Igarashi Y., Kitano H., Okazaki T. (2011). Inhibitory effects of dietary glucosylceramides on squamous cell carcinoma of the head and neck in NOD/SCID mice. Int. J. Clin. Oncol..

[347] Brahmbhatt V.V., Hsu F.F., Kao J.L., Frank E.C., Ford D.A. (2007). Novel carbonyl and nitrile products from reactive chlorinating species attack of lysosphingolipid. Chem. Phys. Lipids.

[348] Semenkova G.N., Amaegberi N.V., Lisovskaya A.G., Pinchuk S.V., Poleshko C., Shadyro O.I. (2023). 2-Hexadecenal Regulates ROS Production and Induces Apoptosis in Polymorphonuclear Leucocytes. Cell Biochem. Biophys..

[349] Stoffel W. (1973). Sphingosine metabolism and its link to phospholipid biosynthesis. Mol. Cell. Biochem..

[350] Kitamura T., Seki N., Kihara A. (2017). Phytosphingosine degradation pathway includes fatty acid α-oxidation reactions in the endoplasmic reticulum. Proc. Natl. Acad. Sci. USA.

[351] Ebenezer D.L., Fu P., Ramchandran R., Ha A.W., Putherickal V., Sudhadevi T., Harijith A., Schumacher F., Kleuser B., Natarajan V. (2020). S1P and plasmalogen derived fatty aldehydes in cellular signaling and functions. Biochim. Biophys. Acta Mol. Cell Biol. Lipids.

[352] Jarugumilli G.K., Choi J.R., Chan P., Yu M., Sun Y., Chen B., Niu J., DeRan M., Zheng B., Zoeller R. (2018). Chemical Probe to Identify the Cellular Targets of the Reactive Lipid Metabolite 2-trans-Hexadecenal. ACS Chem. Biol..

[353] Rizzo W.B. (2007). Sjogren-Larsson syndrome: Molecular genetics and biochemical pathogenesis of fatty aldehyde dehydrogenase deficiency. Mol. Genet. Metab..

[354] Rizzo W.B., S’Aulis D., Dorwart E., Bailey Z. (2022). Sjogren-Larsson syndrome: A biochemical rationale for using aldehyde-reactive therapeutic agents. Mol. Genet. Metab. Rep..

[355] Van Overloop H., Van der Hoeven G., Van Veldhoven P.P. (2005). N-acyl migration in ceramides. J. Lipid Res..

[356] Zhou Y., Park H., Kim P., Jiang Y., Costello C.E. (2014). Surface oxidation under ambient air—Not only a fast and economical method to identify double bond positions in unsaturated lipids but also a reminder of proper lipid processing. Anal. Chem..

[357] Couto D., Santinha D., Melo T., Ferreira-Fernandes E., Videira R.A., Campos A., Fardilha M., Domingues P., Domingues M.R. (2015). Glycosphingolipids and oxidative stress: Evaluation of hydroxyl radical oxidation of galactosyl and lactosylceramides using mass spectrometry. Chem. Phys. Lipids.

[358] Jutanom M., Kato S., Yamashita S., Toda M., Kinoshita M., Nakagawa K. (2023). Analysis of oxidized glucosylceramide and its effects on altering gene expressions of inflammation induced by LPS in intestinal tract cell models. Sci. Rep..

[359] Lee W.K., Lam T.K.Y., Tang H.C., Ho T.C., Wan H.T., Wong C.K.C. (2023). PFOS-elicited metabolic perturbation in liver and fatty acid metabolites in testis of adult mice. Front. Endocrinol..

[360] Kofeler H.C., Ahrends R., Baker E.S., Ekroos K., Han X., Hoffmann N., Holcapek M., Wenk M.R., Liebisch G. (2021). Recommendations for good practice in MS-based lipidomics. J. Lipid Res..

[361] Wijesinghe D.S., Allegood J.C., Gentile L.B., Fox T.E., Kester M., Chalfant C.E. (2010). Use of high performance liquid chromatography-electrospray ionization-tandem mass spectrometry for the analysis of ceramide-1-phosphate levels. J. Lipid Res..

[362] Berdyshev E.V., Gorshkova I.A., Garcia J.G., Natarajan V., Hubbard W.C. (2005). Quantitative analysis of sphingoid base-1-phosphates as bisacetylated derivatives by liquid chromatography—Tandem mass spectrometry. Anal. Biochem..

[363] Frej C., Andersson A., Larsson B., Guo L.J., Norstrom E., Happonen K.E., Dahlback B. (2015). Quantification of sphingosine 1-phosphate by validated LC-MS/MS method revealing strong correlation with apolipoprotein M in plasma but not in serum due to platelet activation during blood coagulation. Anal. Bioanal. Chem..

[364] Gowda S.G.B., Ikeda K., Arita M. (2018). Facile determination of sphingolipids under alkali condition using metal-free column by LC-MS/MS. Anal. Bioanal. Chem..

[365] Morano C., Zulueta A., Caretti A., Roda G., Paroni R., Dei Cas M. (2022). An Update on Sphingolipidomics: Is Something Still Missing? Some Considerations on the Analysis of Complex Sphingolipids and Free-Sphingoid Bases in Plasma and Red Blood Cells. Metabolites.

[366] Skotland T., Ekroos K., McDonald J., Ahrends R., Liebisch G., Sandvig K. (2024). Pitfalls in lipid mass spectrometry of mammalian samples—A brief guide for biologists. Nat. Rev. Mol. Cell Biol..

[367] Kopczynski D., Ejsing C.S., McDonald J.G., Bamba T., Baker E.S., Bertrand-Michel J., Brugger B., Coman C., Ellis S.R., Garrett T.J. (2024). The lipidomics reporting checklist a framework for transparency of lipidomic experiments and repurposing resource data. J. Lipid Res..

[368] Torta F., Hoffmann N., Burla B., Alecu I., Arita M., Bamba T., Bennett S.A.L., Bertrand-Michel J., Brugger B., Cala M.P. (2024). Concordant inter-laboratory derived concentrations of ceramides in human plasma reference materials via authentic standards. Nat. Commun..

[369] Wang M., Wang C., Han X. (2017). Selection of internal standards for accurate quantification of complex lipid species in biological extracts by electrospray ionization mass spectrometry—What, how and why?. Mass Spectrom. Rev..

[370] Thudichum J.L.W. (1962). A Treatise on the Chemical Constitution of the Brain.

